# Supplementary material for: A countercurrent microflow strategy for simultaneous high selectivity and conversion in aromatic nitration
Source: Nat Commun. 2026 Feb 20;17:2990. doi: 10.1038/s41467-026-69902-2 (PMC13036075; doi:10.1038/s41467-026-69902-2)
Supplement: Supplementary file 1 — Supplementary Information [file 41467_2026_69902_MOESM1_ESM.pdf]

Supplementary Information

A Countercurrent Microflow Strategy for Simultaneous High Selectivity and Conversion in Aromatic Nitration

Jing Song[1], Yongqi Pan[1], Ruobing Xin[1], Zifei Yan[1], Tianyao Tang[1], Kai Wang[1], Yujun Wang[1], Jian Deng[1], Guangsheng Luo[1*]

[1] State Key Laboratory of Chemical Engineering and Low-Carbon Technology, Department of Chemical Engineering, Tsinghua University, Beijing 100084, China

[*]Corresponding author. E-mail: gsluo@tsinghua.edu.cn

Table of Contents

Supplementary Note 1: Derivation of Eq. (1) 4
Supplementary Note 2: Derivation of Eq. (2) 5
Supplementary Note 3: Reaction network of toluene nitration 7
Supplementary Note 4: Performance of toluene nitration in the first-stage microreactor 8
4.1 Effect of flow rate ratio and temperature on reaction performance 8
4.2 Effect of residence time on reaction performance 9
Supplementary Note 5: Application of the microreaction strategy in benzene nitration 10
5.1 Performance of benzene nitration 10
5.2 Thermodynamic properties of benzene and mixed acid system 12
Supplementary Note 6: Application of the microreaction strategy in chlorobenzene nitration 13
6.1 Performance of chlorobenzene nitration 13
6.2 Thermodynamic properties of benzene and mixed acid system 16
Supplementary Note 7: Common laws of aromatic and mixed acid nitration systems 17
7.1 Common laws of reaction kinetics 17
7.2 Common laws of thermodynamics 17
Supplementary Note 8: Product composition analysis 21
8.1 Gas chromatography-mass spectrometry (GC-MS) analysis of reaction products in the batch nitration system 21
8.2 Gas chromatography-mass spectrometry (GC-MS) analysis of reaction products in the two-stage countercurrent nitration system 30
8.3 Ultra-high performance liquid chromatography-mass spectrometry (UPLC-MS) analysis of reaction products in the two-stage countercurrent nitration system 31
Supplementary Note 9: Analysis of Hatta number under actual operating conditions 35
Supplementary Note 10: Long-term stability test of the two-stage countercurrent microreaction system 37
Supplementary Note 11: Comparison of operating conditions and performance with literature reports 39
Supplementary Note 12: Determination of the optimal H_2SO_4/HNO_3 dilution

window 41
References 43

Supplementary Note 1: Derivation of Eq. (1)

$$r = kc_{\mathrm{ar}}c_{\mathrm{N}} \tag{S1}$$

$$r = -\frac{dc_{\mathrm{ar}}}{dt} \tag{S2}$$

$$c_{\mathrm{ar}} = c_{\mathrm{ar}}^{0}(1 - x_{\mathrm{ar}}) \tag{S3}$$

$$c_{\mathrm{N}} = c_{\mathrm{N}}^{0} - c_{\mathrm{ar}}^{0}x_{\mathrm{ar}} \tag{S4}$$

$$\frac{dx_{\mathrm{ar}}}{dt} = k(1 - x_{\mathrm{ar}})(c_{\mathrm{N}}^{0} - c_{\mathrm{ar}}^{0}x_{\mathrm{ar}}) \tag{S5}$$

where x is conversion, c is the concentration of substance, k is the observed reaction rate constant, t is the residence time. The superscript 0 represents the initial moment of the reaction, and the subscripts ar and N represent aromatic and HNO_3, respectively. By integrating Eq. (S5), we can obtain:

$$\ln\left[\frac{\frac{c_{\mathrm{N}}^{0}}{c_{\mathrm{ar}}^{0}} - x_{\mathrm{ar}}}{\frac{c_{\mathrm{N}}^{0}}{c_{\mathrm{ar}}^{0}}(1 - x_{\mathrm{ar}})}\right] = k\left(c_{\mathrm{N}}^{0} - c_{\mathrm{ar}}^{0}\right)t \tag{S6}$$

$$x_{\mathrm{ar}} = \frac{\frac{c_{\mathrm{N}}^{0}}{c_{\mathrm{ar}}^{0}}\left(1 - e^{k(c_{\mathrm{N}}^{0} - c_{\mathrm{ar}}^{0})t}\right)}{1 - \frac{c_{\mathrm{N}}^{0}}{c_{\mathrm{ar}}^{0}}e^{k(c_{\mathrm{N}}^{0} - c_{\mathrm{ar}}^{0})t}} \tag{S7}$$

Supplementary Note 2: Derivation of Eq. (2)

In the two-stage countercurrent microflow mode, there is still co-current microflow in each microreactor, and the reaction process conforms to the second-order reaction kinetics. The total residence time is equal to the sum of the residence times in the two microreactors. Assume that the initial molar concentration of aromatic in the first-stage microreactor is c_{ar}^{0}. The initial molar concentration of HNO_3 in the second-stage microreactor is c_{N}^{0}. The conversion rate of toluene in the first-stage microreactor is $x_{\mathrm{ar},1}$. The residual concentration of aromatic at the outlet of the first-stage microreactor is $c_{\mathrm{ar}}^{0}(1-x_{\mathrm{ar},1})$. Therefore, the initial concentration of aromatic in the second-stage microreactor is $c_{\mathrm{ar}}^{0}(1-x_{\mathrm{ar},1})$. In the second-stage microreactor, the reaction rate equation is:

$$r=k\left[c_{\mathrm{ar}}^{0}(1-x_{\mathrm{ar},1})(1-x_{\mathrm{ar},2})\right]\left[c_{\mathrm{N}}^{0}-c_{\mathrm{ar}}^{0}(1-x_{\mathrm{ar},1})x_{\mathrm{ar},2}\right] \tag{S8}$$

$$r=-\frac{\mathrm{d}c_{\mathrm{ar},2}}{\mathrm{d}t}=-\frac{\mathrm{d}\left[c_{\mathrm{ar}}^{0}(1-x_{\mathrm{ar},1})(1-x_{\mathrm{ar},2})\right]}{\mathrm{d}t} \tag{S9}$$

$$c_{\mathrm{ar},0}(1-x_{\mathrm{ar},1})\frac{\mathrm{d}x_{\mathrm{ar},2}}{\mathrm{d}t}=k\left[c_{\mathrm{ar}}^{0}(1-x_{\mathrm{ar},1})(1-x_{\mathrm{ar},2})\right]\left[c_{\mathrm{N}}^{0}-c_{\mathrm{ar}}^{0}(1-x_{\mathrm{ar},1})x_{\mathrm{ar},2}\right] \tag{S10}$$

$$\frac{c_{\mathrm{ar}}^{0}(1-x_{\mathrm{ar},1})}{\left[c_{\mathrm{ar}}^{0}(1-x_{\mathrm{ar},1})(1-x_{\mathrm{ar},2})\right]\left[c_{\mathrm{N}}^{0}-c_{\mathrm{ar}}^{0}(1-x_{\mathrm{ar},1})x_{\mathrm{ar},2}\right]}\mathrm{d}x_{\mathrm{ar},2}=k\mathrm{d}t \tag{S11}$$

By integrating Eq. (S11), the residence time in the second-stage microreactor t_2 can be obtained:

$$t_2=\frac{1}{k\left[c_{\mathrm{ar}}^{0}(1-x_{\mathrm{ar},1})-c_{\mathrm{N}}^{0}\right]}\ln\left[\frac{c_{\mathrm{N}}^{0}(1-x_{\mathrm{ar}})}{c_{\mathrm{N}}^{0}-c_{\mathrm{ar}}^{0}(1-x_{\mathrm{ar},1})x_{\mathrm{ar}}}\right] \tag{S12}$$

where x_{ar} is the total target aromatic conversion.

According to the design of two-stage countercurrent, the initial concentration of HNO_3 entering the first-stage microreactor is $c_{\mathrm{N}}^{0}-c_{\mathrm{ar}}^{0}(1-x_{\mathrm{ar},1})$. The initial concentration of aromatic entering the first-stage microreactor is c_{ar}^{0}. Therefore, in the first-stage

microreactor, the reaction rate equation is:

$$r = k\left[c_{\mathrm{ar}}^{0}(1-x_{\mathrm{ar}})\right]\left[c_{\mathrm{N}}^{0} - c_{\mathrm{ar}}^{0}(1-x_{\mathrm{ar,1}}) - c_{\mathrm{ar}}^{0}x_{\mathrm{ar}}\right] \tag{S13}$$

$$-\frac{\mathrm{d}c_{\mathrm{ar,1}}}{\mathrm{d}t} = -\frac{\mathrm{d}\left[c_{\mathrm{ar}}^{0} - c_{\mathrm{ar}}^{0}x_{\mathrm{ar}}\right]}{\mathrm{d}t} = c_{\mathrm{ar}}^{0}\frac{\mathrm{d}x_{\mathrm{ar}}}{\mathrm{d}t} \tag{S14}$$

where the range of x_{ar} is $[0, x_{\mathrm{ar,\,1}}]$.

$$c_{\mathrm{ar}}^{0}\frac{\mathrm{d}x_{\mathrm{ar}}}{\mathrm{d}t} = kc_{\mathrm{ar}}^{0}\left(1-x_{\mathrm{ar}}\right)\left[c_{\mathrm{N}}^{0} - c_{\mathrm{ar}}^{0}\left(1-x_{\mathrm{ar,1}}\right) - c_{\mathrm{ar}}^{0}x_{\mathrm{ar}}\right] \tag{S15}$$

$$\left(1-x_{\mathrm{ar}}\right)\left[c_{\mathrm{N}}^{0} - c_{\mathrm{ar}}^{0}\left(1-x_{\mathrm{ar,1}}\right) - c_{\mathrm{ar}}^{0}x_{\mathrm{ar}}\right]\mathrm{d}x_{\mathrm{ar}} = k\mathrm{d}t \tag{S16}$$

By integrating Eq. (S16), the residence time in the first-stage microreactor t_1 can be obtained:

$$t_1 = \frac{1}{k\left[c_{\mathrm{N}}^{0} - c_{\mathrm{ar}}^{0}(1-x_{\mathrm{ar,1}}) - c_{\mathrm{ar}}^{0}\right]}\ln\left[\frac{c_{\mathrm{ar}}^{0}(1-x_{\mathrm{ar}})}{c_{\mathrm{ar}}^{0} - \left[c_{\mathrm{N}}^{0} - c_{\mathrm{ar}}^{0}(1-x_{\mathrm{ar,1}})\right]x_{\mathrm{ar}}}\right] \tag{S17}$$

In summary, the residence time of the tow-stage countercurrent microflow tt can be obtained:

$$\begin{aligned} t_{\mathrm{t}} = &\frac{1}{k\left[c_{\mathrm{N}}^{0} - c_{\mathrm{ar}}^{0}(1-x_{\mathrm{ar,1}}) - c_{\mathrm{ar}}^{0}\right]}\ln\left[\frac{c_{\mathrm{ar}}^{0}(1-x_{\mathrm{ar}})}{c_{\mathrm{ar}}^{0} - \left[c_{\mathrm{N}}^{0} - c_{\mathrm{ar}}^{0}(1-x_{\mathrm{ar,1}})\right]x_{\mathrm{ar}}}\right] \\ &+ \frac{1}{k\left[c_{\mathrm{ar}}^{0}(1-x_{\mathrm{ar,1}}) - c_{\mathrm{N}}^{0}\right]}\ln\left[\frac{c_{\mathrm{N}}^{0}(1-x_{\mathrm{ar}})}{c_{\mathrm{N}}^{0} - c_{\mathrm{ar}}^{0}(1-x_{\mathrm{ar,1}})x_{\mathrm{ar}}}\right] \end{aligned} \tag{S18}$$

Supplementary Note 3: Reaction network of toluene nitration

The mononitration reaction of toluene with mixed acid can produce *o*-nitrotoluene, *m*-nitrotoluene and *p*-nitrotoluene. The nitration reaction of *o*-nitrotoluene with mixed acid can produce 2,4-dinitrotoluene (2,4-DNT) and 2,6-dinitrotoluene (2,6-DNT). The nitration reaction of *p*-nitrotoluene with mixed acid can produce 2,4-DNT. Both 2,4-DNT and 2,6-DNT can further undergo nitration to produce 2,4,6-trinitrotoluene (TNT). Therefore, for the mononitration reaction of toluene, the reactions that produce DNT and TNT are both the over-nitration side reactions. The reaction network of toluene nitration is shown in Figure S1.

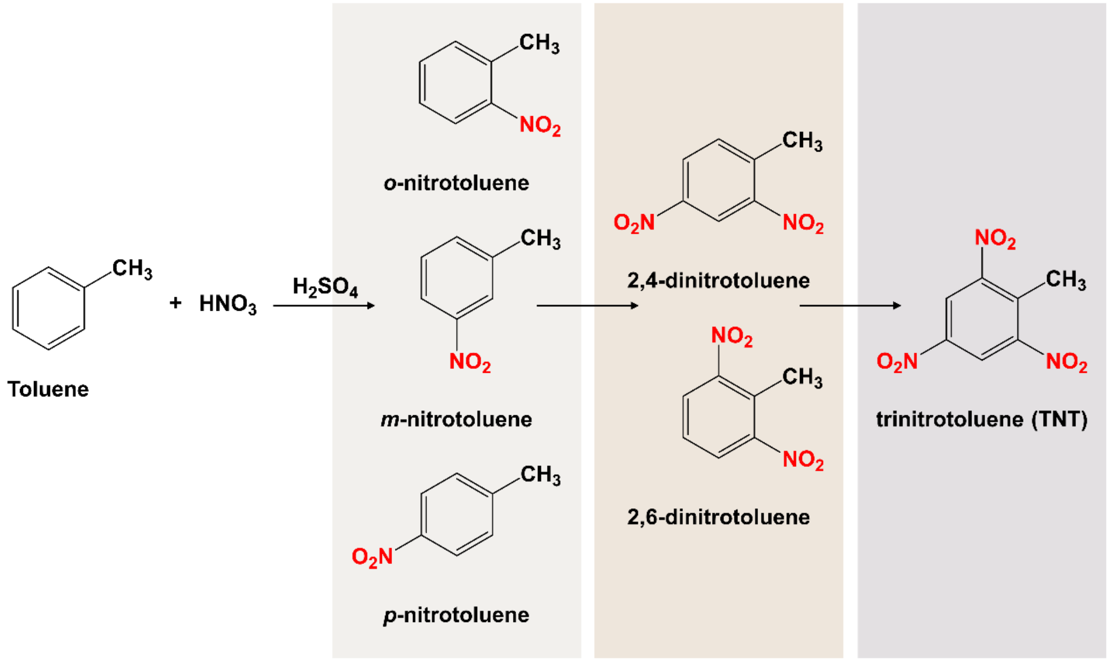

Figure S1. Reaction network of toluene nitration with mixed acid.

Supplementary Note 4: Performance of toluene nitration in the first-stage microreactor

4.1 Effect of flow rate ratio and temperature on reaction performance

In the first-stage microreactor, the initial molar ratio of toluene to HNO_3 was 1:0.501. Therefore, the challenge of controlling over-nitration side reactions is significantly reduced compared to the second-stage microreactor. The effect of aqueous-to-organic flow rate ratio and temperature on the over-nitration behavior is shown in Figure S2. As shown in Figure S2(a), decreasing the dosage of H_2SO_4 increases the initial concentration of HNO_3, thereby accelerating the reaction rate. As shown in Figure S2(b), when $1 < Q_a/Q_o < 3$, a lower H_2SO_4 dosage enhances the dilution effect of H_2O generated from the main reaction, effectively suppressing the over-nitration side reaction in-situ. When $Q_a/Q_o > 3$, the reaction rate is slower and the interface temperature rise of the system is reduced, thereby reducing the occurrence of the over-nitration side reaction due to the increase in system temperature. These observations align with previously reported trends, highlighting the intrinsic trade-off effect between spatiotemporal conversion rate and selectivity.

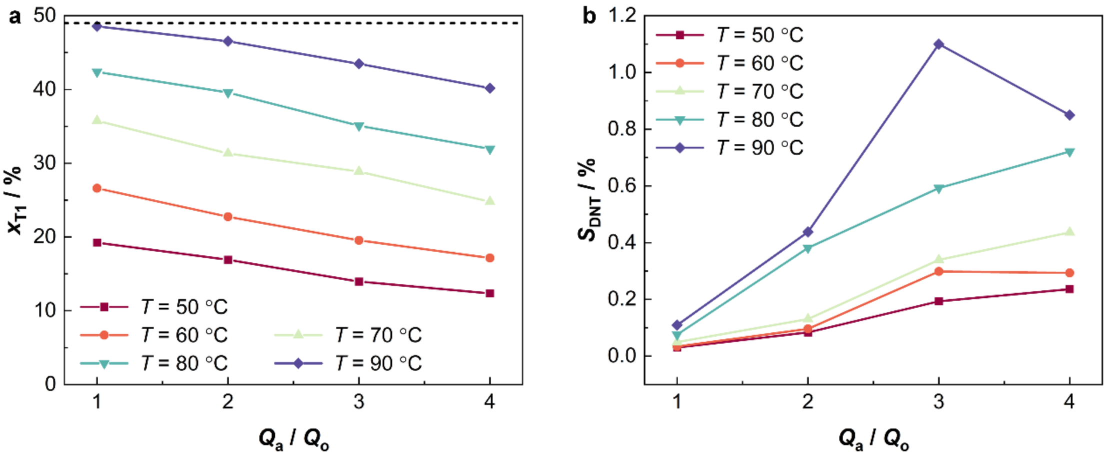

Figure S2. Effect of Q_a/Q_o and temperature on the (a) conversion of toluene and (b) selectivity of over-nitration side reaction in the first-stage microreactor.

4.2 Effect of residence time on reaction performance

The effect of residence time and temperature on the over-nitration behavior is shown in Figure S3. As shown in Figure S3(a), the reaction rate decreases with increasing residence time, consistent with second-order reaction kinetics. As shown in Figure S3(b), in the first-stage microreactor, the selectivity of over-nitration side reaction decreases as residence time increases. This shows that as the reaction proceeds, the concentration of H_2SO_4 decreases, thereby enhancing the inhibitory effect on over-nitration side reaction.

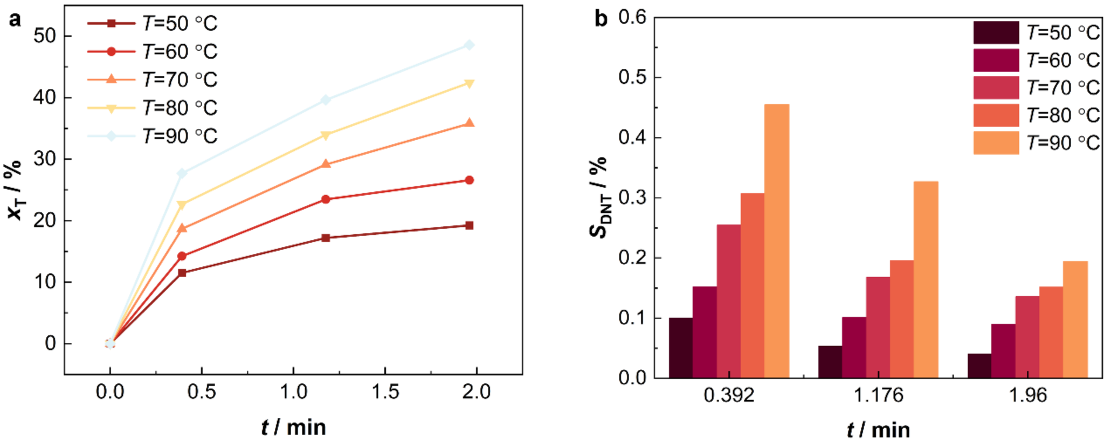

Figure S3. Effect of residence time on the (a) conversion of toluene and (b) selectivity of over-nitration side reaction in the first-stage microreactor.

Supplementary Note 5: Application of the microreaction strategy in benzene nitration

5.1 Performance of benzene nitration

The reaction network of benzene nitration is shown in Figure S4. The reaction mechanism of the benzene nitration with the mixed acid is the same as that of toluene nitration. But the reaction network of benzene nitration is simpler than that of toluene nitration. Moreover, based on the reaction mechanism of electrophilic substitution, the nitration activity of benzene is significantly lower than that of toluene. Therefore, the trade-off effect of benzene nitration is lower than that of toluene nitration.

The initial molar ratio of benzene to HNO_3 is still set to 1:1.01. Therefore, according to the design principle of the two-stage countercurrent microflow mode, the process design of benzene nitration is shown in Figure S5.

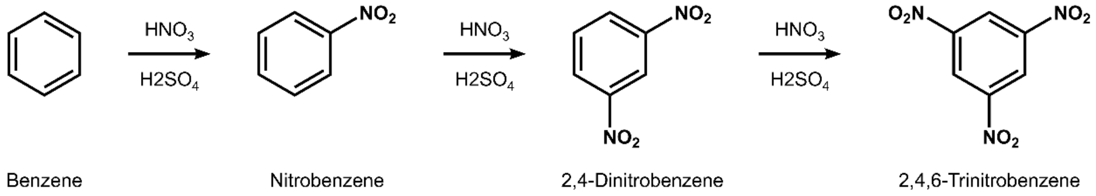

Figure S4. Reaction network of benzene nitration with mixed acid.

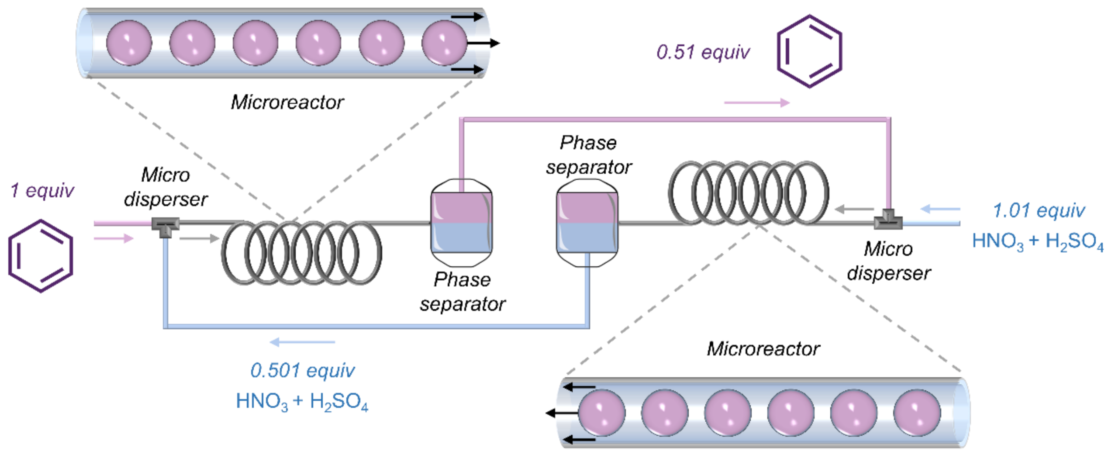

Figure S5. process design of benzene nitration.

The composition of the products of benzene nitration was detected by gas

chromatography (GC-2014C, SHIMADZU, China). The detection method is shown in Table S1.

Table S1. Gas chromatography detection method for benzene nitration system

Instrument	Chromatographic column	Carrier gas	Split Ratio	Total flow rate	Pressure
GC-2014C, SHIMADZU	AE·SE-54 30 m*0.32 mm*0.33 μm	N_2	30:1	65 mL/min	72.3 kPa

Detector	Injection volume	Heating gradient curve
FID hydrogen flame detector	20 μL	*T*=90 °C. Temperature holding time: 2 min. Heating rate: 10 °C/min;<br>*T*=130 °C. Temperature holding time: 2 min. Heating rate: 20 °C/min;<br>*T*=200 °C. Temperature holding time: 2 min. Heating rate: 20 °C/min;<br>*T*=230 °C. Temperature holding time: 3 min.

The performance of benzene nitration using the proposed microreaction strategy is shown in Table S2. Compared to literature reports[1], this approach achieves higher benzene conversion and nitrobenzene selectivity with less HNO_3 dosage. Notably, over-nitration by-products are nearly undetectable in the final product.

Table S2. Performance of benzene nitration based on different reaction modes

Reaction mode	T / °C	W_{H2SO4} / %	M	Q_a/Q_o	t / min	x_B / %	S_{DNB} / %
Two-stage countercurrent microflow	90	75	1.01	1	2.0	> 99.9	> 99.9
Single-stage co-current microflow[1]	70	71.5	1.05	—	2.2	99.5	99.6

5.2 Thermodynamic properties of benzene and mixed acid system

The solubility of benzene and nitrobenzene in H_2SO_4 was further studied, as shown in Figure S6. Benzene is a typical non-polar molecule, while concentrated sulfuric acid is a highly polar strong acid. As shown in Figure S6, benzene is completely insoluble in 70% ~ 85% H_2SO_4. In contrast, the introduction of a nitro group significantly increases the molecular polarity of nitrobenzene, thereby enhancing its solubility in H_2SO_4.

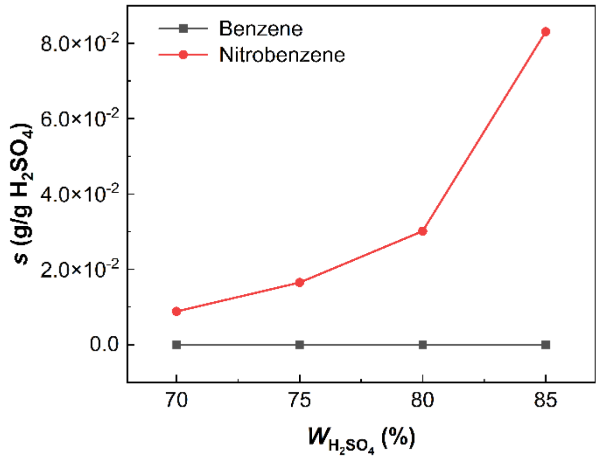

Figure S6. Solubility of benzene and nitrobenzene in H_2SO_4 of different concentrations.

Consistent with the toluene nitration system, a decrease in H_2SO_4 concentration significantly reduces the solubility difference between benzene and nitrobenzene. Therefore, at low H_2SO_4 dosage, the water produced by benzene nitration will also significantly reduce the solubility of nitrobenzene in sulfuric acid, thereby avoiding the occurrence of over-nitration side reactions. Critically, the implementation of the two-stage countercurrent microflow mode effectively compensates for the reduced benzene nitration rate resulting from lower H_2SO_4 concentrations. Therefore, the trade-off effect of benzene nitration was effectively overcome by adopting the novel microreaction strategy.

Supplementary Note 6: Application of the microreaction strategy in chlorobenzene nitration

6.1 Performance of chlorobenzene nitration

The reaction network of chlorobenzene nitration is illustrated in Figure S7. Due to the electron-withdrawing characteristic of the –Cl substituent, the electrophilic reactivity of the aromatic ring is diminished, leading to a slower nitration rate compared to benzene nitration. The microreaction strategy developed in this work is also applied to chlorobenzene to overcome the inherent trade-off between spatiotemporal conversion rate and selectivity. The corresponding reaction process is depicted in Figure S8.

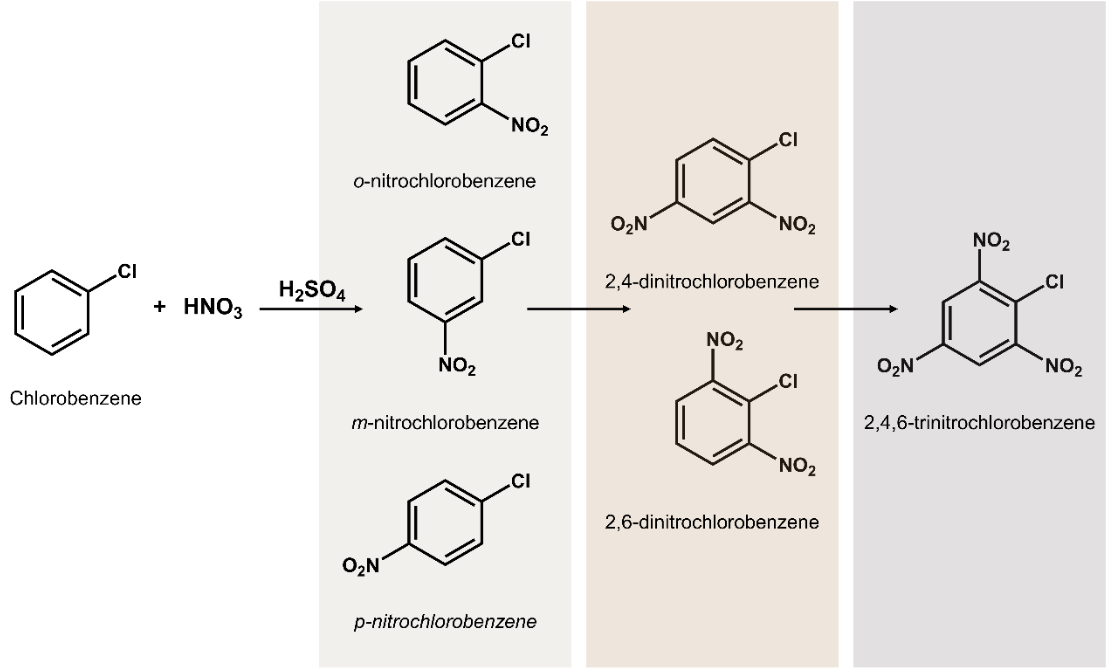

Figure S7. Reaction network of chlorobenzene nitration with mixed acid.

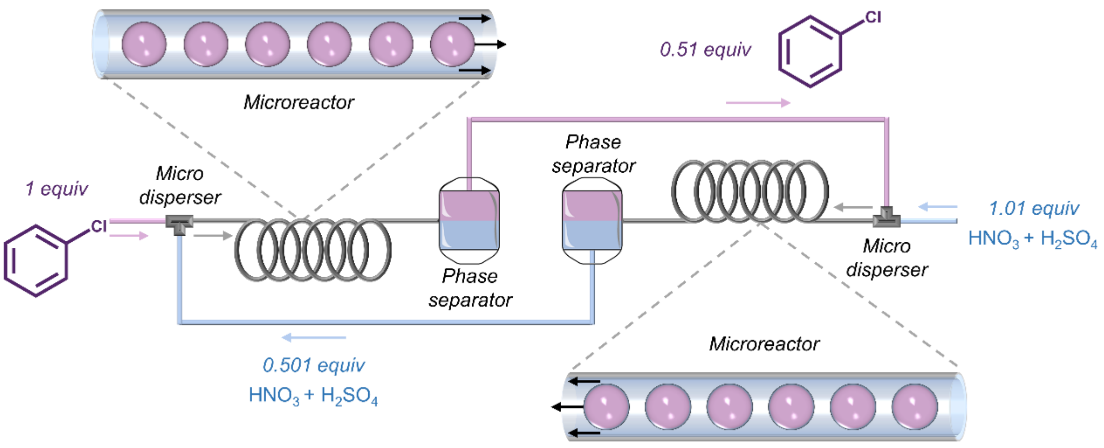

Figure S8. process design of chlorobenzene nitration.

The composition of the products of chlorobenzene nitration was also detected by gas chromatography (GC-2014C, SHIMADZU, China). The detection method is shown in Table S1.

Table S3. Gas chromatography detection method for chlorobenzene nitration system

Instrument	Chromatographic column	Carrier gas	Split Ratio	Total flow rate	Pressure
GC-2014C, SHIMADZU	AE·SE-54 30 m*0.32 mm*0.33 μm	N_2	30:1	65 mL/min	72.3 kPa

Detector	Injection volume	Heating gradient curve
FID hydrogen flame detector	20 μL	*T*=90 °C. Temperature holding time: 2 min. Heating rate: 10 °C/min; *T*=130 °C. Temperature holding time: 2 min. Heating rate: 20 °C/min; *T*=200 °C. Temperature holding time: 2 min. Heating rate: 20 °C/min; *T*=230 °C. Temperature holding time: 3 min.

The performance of chlorobenzene nitration using the proposed microreaction strategy is shown in Table S4. Compared to literature reports, this approach achieves higher chlorobenzene conversion and nitrochlorobenzene selectivity with less HNO_3 dosage. The over-nitration by-products are not undetectable in the final product.

Table S4. Performance of chlorobenzene nitration based on different reaction modes

Reaction mode	T / °C	W_{H2SO4} / %	M	Q_a/Q_o	t / min	x_B / %	S_{NCB} / %
Two-stage countercurrent microflow	90	80	1.01	1	4	> 99.9	99.99
Single-stage co-current microflow[2]	90	90	1.2	—	1	96.39	97.47

6.2 Thermodynamic properties of benzene and mixed acid system

The solubility of chlorobenzene and nitrochlorobenzene in H_2SO_4 was further studied, as shown in Figure S9. Chlorobenzene is more polar than benzene. As shown in Figure S9, chlorobenzene can be partially dissolved in concentrated H_2SO_4. Nitrochlorobenzene is more polar, and its solubility in sulfuric acid solution is always higher than that of chlorobenzene. Consistent with the nitration system of toluene and benzene, the solubility difference between nitrochlorobenzene and chlorobenzene is significantly reduced as the concentration of H_2SO_4 decreases. This indicates that the product inhibition mechanism driven by thermodynamic property regulation is also applicable to the nitration of chlorobenzene.

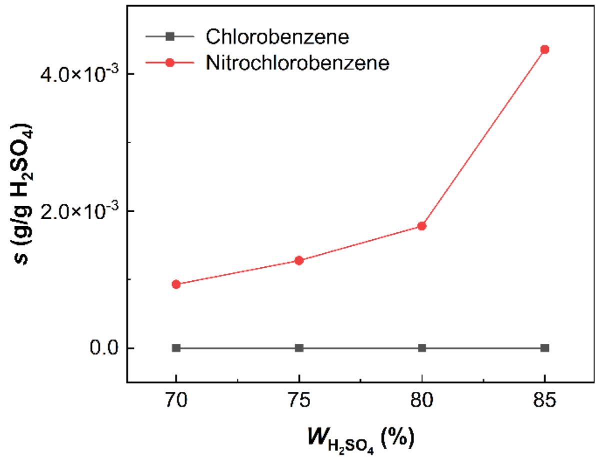

Figure S9. Solubility of chlorobenzene and nitrochlorobenzene in H_2SO_4 of different concentrations.

Supplementary Note 7: Common laws of aromatic and mixed acid nitration systems

7.1 Common laws of reaction kinetics

The two-stage countercurrent microflow mode is completely based on the kinetics of the second-order reaction. Its advantages in improving the total spatiotemporal conversion rate and reducing the system interface temperature rise are applicable to all second-order reactions. Combined with the advantages of microreactors in intensifying the transport process, the system temperature can be controlled more accurately. The aromatic nitration reaction mechanism with mixed acid follows the electrophilic substitution reaction mechanism, and the reaction order is second-order. Therefore, the two-stage countercurrent mode is applicable to the nitration reaction of aromatics.

7.2 Common laws of thermodynamics

The introduction of nitro ($-NO_2$) will enhance the polarity of the benzene ring, which is a common law in aromatic nitration process. This means that the solubility of aromatic nitration products in H_2SO_4 is always higher than that of aromatics. Molecular simulation was used to prove this conclusion at the molecular level.

All molecular simulations were performed using density functional theory (DFT). The initial geometries of *p*-nitrotoluene–solvent complexes were optimized using the semiempirical PM6-D3H4 method. Electronic structure analyses and electron density difference calculations were subsequently carried out using the B97-3c composite method. To mimic solvent environments, p-nitrotoluene was explicitly coordinated with either sulfuric acid or water molecules. Electron density difference maps were generated by subtracting the electron densities of the isolated fragments from that of the optimized complex. Hydrogen bonds were identified based on geometric criteria of O···O distance < 3.5 Å and O–H···O angle > 150°. Gibbs free energy change (ΔG) was obtained from frequency calculations and used to evaluate equilibrium constants for

nitronium ion (NO_2^+) formation under different temperatures and acid compositions.

Figure S10 shows the optimized structures and electron density difference maps of *p*-nitrotoluene interacting with H_2SO_4 and H_2O molecules. In H_2SO_4, the nitro group (-NO_2) of *p*-nitrotoluene forms strong and directional hydrogen bonds with the O-H groups of H_2SO_4 (Figure S10a). Significant electron density accumulation is observed between the nitro oxygen atoms and the acidic protons of H_2SO_4, confirming the formation of stabilizing hydrogen bonds. In contrast, other regions of the aromatic ring show negligible interaction with H_2SO_4 molecules. In H_2O environments, *p*-nitrotoluene can also form hydrogen bonds between the nitro group and H_2O molecules (Figure S10b). However, the O···O distances are noticeably longer and the electron density redistribution is much weaker compared with H_2SO_4, indicating weaker hydrogen bonding interactions. The equilibrium distance between *p*-nitrotoluene and H_2SO_4 molecules is consistently shorter than that observed with H_2O molecules. These results demonstrate that the enhanced solubility of nitroaromatics in H_2SO_4 originates from specific nitro-H_2SO_4 hydrogen bonding. This finding proves the general rule in aromatic nitration systems: nitroaromatics are significantly more soluble in H_2SO_4 than aromatics, owing to nitro-specific hydrogen bonding.

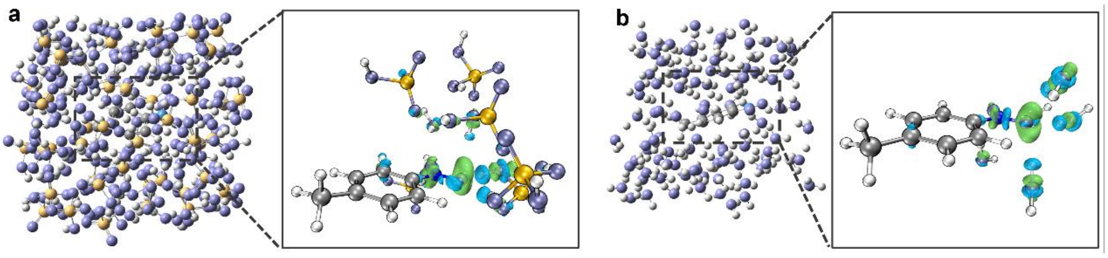

Figure S10. Solvation Structure of *p*-nitrotoluene in H_2SO_4(a) and H_2O(b).

To further understand the effect of H_2O accumulation during nitration, hydrogen-bond networks in H_2SO_4 solutions with different H_2O contents were quantified (Table S5). As H_2O fraction increases, the total number of hydrogen bonds in the system increases. Simultaneously, the hydrogen-bond network gradually transitions from being H_2SO_4-dominated to H_2O-dominated, accompanied by increasing ionization of H_2SO_4. Reduced proton donation capability of H_2SO_4, weakening its ability to stabilize nitroaromatics *via* hydrogen bond. A denser hydrogen bond network, which disfavors

the incorporation of nonpolar organic molecules. As a result, the energetic cost of disrupting the hydrogen bond network to solvate nitroaromatics increases with H_2O content, making their dissolution into the acid phase thermodynamically unfavorable.

Table S5. Evolution of hydrogen bond network in H2SO4-H2O mixtures at different H2O molar fractions

H_2O molar fraction (%)	Number of O-H bonds in H_2SO_4	Number of O-H bonds in H_2O	Total O-H···O Hydrogen Bonds
0	200	0	53
25	143	57	58
50	82	118	73
75	37	163	76
100	0	200	74

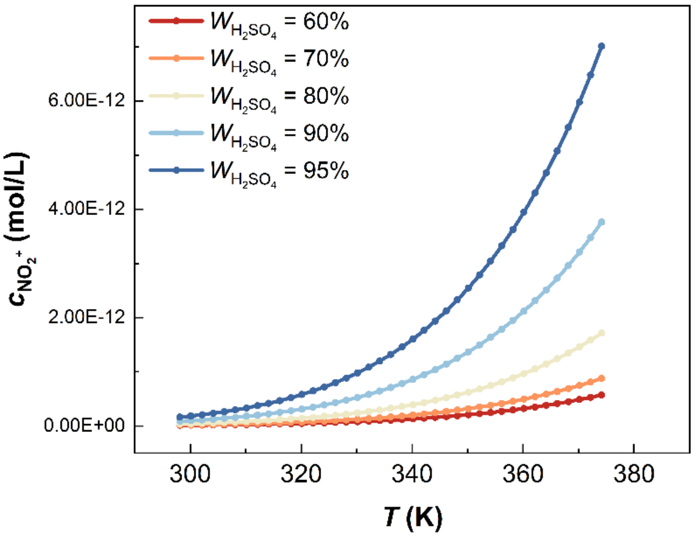

Figure S11. Effect of temperature and H_2SO_4 concentration on NO_2^+ concentration.

Based on the calculated thermodynamic parameters, the equilibrium concentration of NO_2^+ was evaluated as a function of temperature and H_2SO_4 concentration (Figure S11). The results show that increasing temperature promotes NO_2^+ formation, whereas decreasing H_2SO_4 concentration (increasing H_2O content) significantly reduces the equilibrium NO_2^+ concentration. Since NO_2^+ can only exist in the acid phase, these results indicate that in situ H_2O generation during nitration locally dilutes H_2SO_4 at the droplet interface, thereby reducing the activity of NO_2^+. This not only thermodynamically disfavors the dissolution of nitroaromatics into the acid phase, but

also kinetically suppresses the rate of over-nitration reaction.

These molecular level trends are fully consistent with the experimentally observed sharp decrease in nitroaromatic solubility with decreasing H_2SO_4 concentration. Therefore, the effect of reducing the dosage of H_2SO_4 to enhance product inhibition is universal for aromatic nitration reactions. The product inhibition mechanism based on the thermodynamic property regulation is widely applicable to suppressing the over-nitration side reaction process in aromatic nitration.

At the same time, the reduction in the dosage of H_2SO_4 will increase the initial concentration of HNO_3, which will accelerate the nitration reaction rate and heat release rate. The two-stage countercurrent microflow mode improves the spatiotemporal conversion rate while reducing the interface temperature rise through process design. Therefore, the coordinated use of thermodynamic and kinetic control methods is a universal method to overcome the trade-off effect between spatiotemporal conversion rate and reaction selectivity in aromatic nitration.

Supplementary Note 8: Product composition analysis

8.1 Gas chromatography-mass spectrometry (GC-MS) analysis of reaction products in the batch nitration system

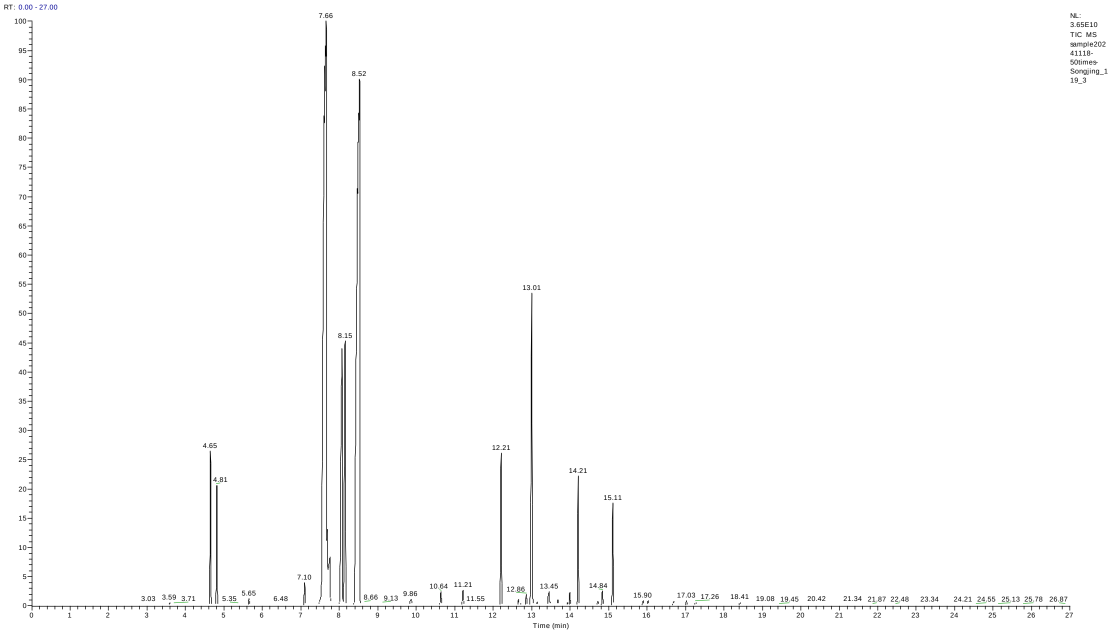

Figure S12. GC-MS spectra of products from the toluene nitration system under batch mode.

Table S6. Performance of chlorobenzene nitration based on different reaction modes

NO.	Retention Time (min)	Substance
1	4.65	benzaldehyde
2	5.65	benzyl alcohol
3	7.66	*o*-nitrotoluene
4	8.15	*m*-nitrotoluene
5	8.52	*p*-nitrotoluene
6	10.64	*p*-nitrobenzaldehyde
7	11.21	*o*-nitrobenzyl alcohol
8	12.21	2,6-dinitrotoluene
9	12.65	2,5-dinitrotoluene
10	12.86	2,3-dinitrotoluene
11	13.01	2,4-dinitrotoluene
12	13.45	3,4-dinitrotoluene
13	13.67	2,4-dinitro-o-cresol
14	14.21	2-methylbenzophenone
15	14.84	3-methylbenzophenone
16	15.11	4-methylbenzophenone

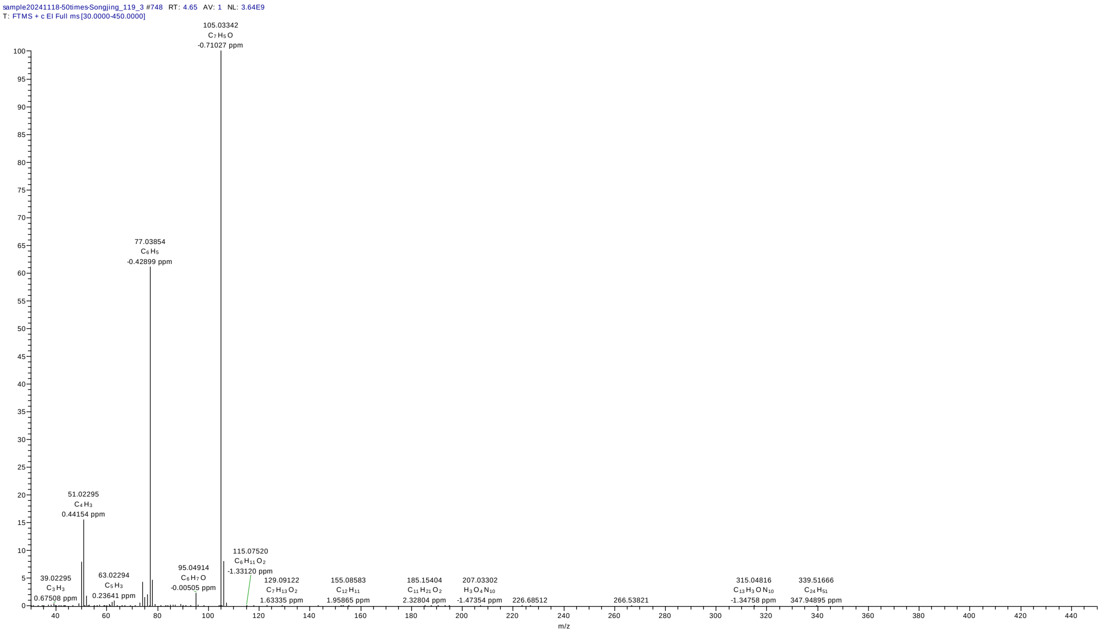

Figure S13. Mass spectrum of benzaldehyde corresponding to the GC peak at *RT* = 4.65 min.

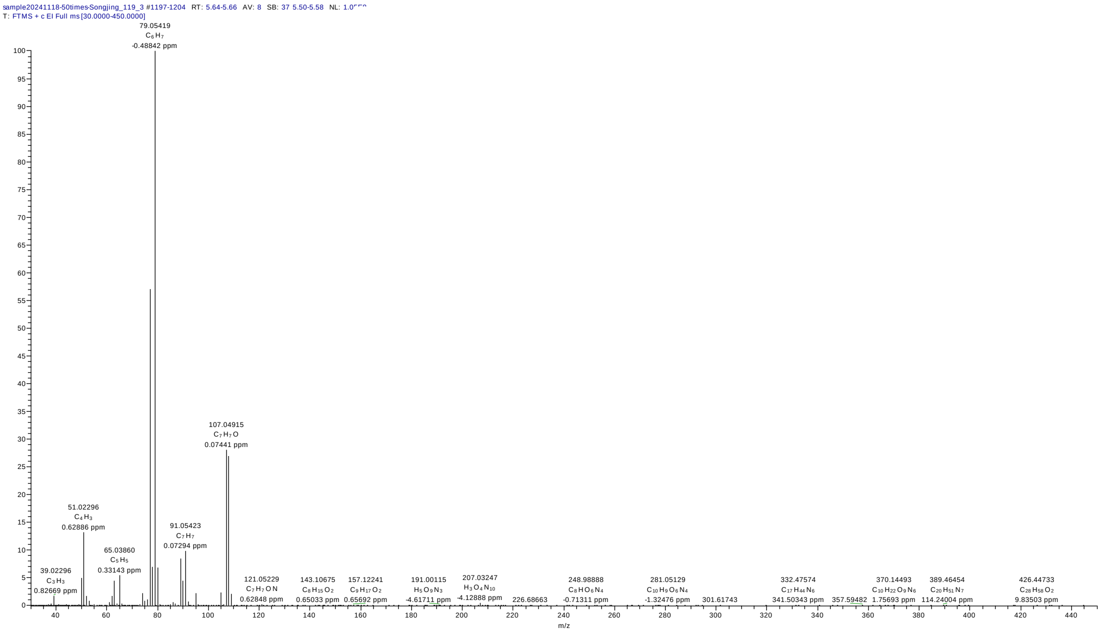

Figure S14. Mass spectrum of benzyl alcohol corresponding to the GC peak at *RT* = 5.65 min.

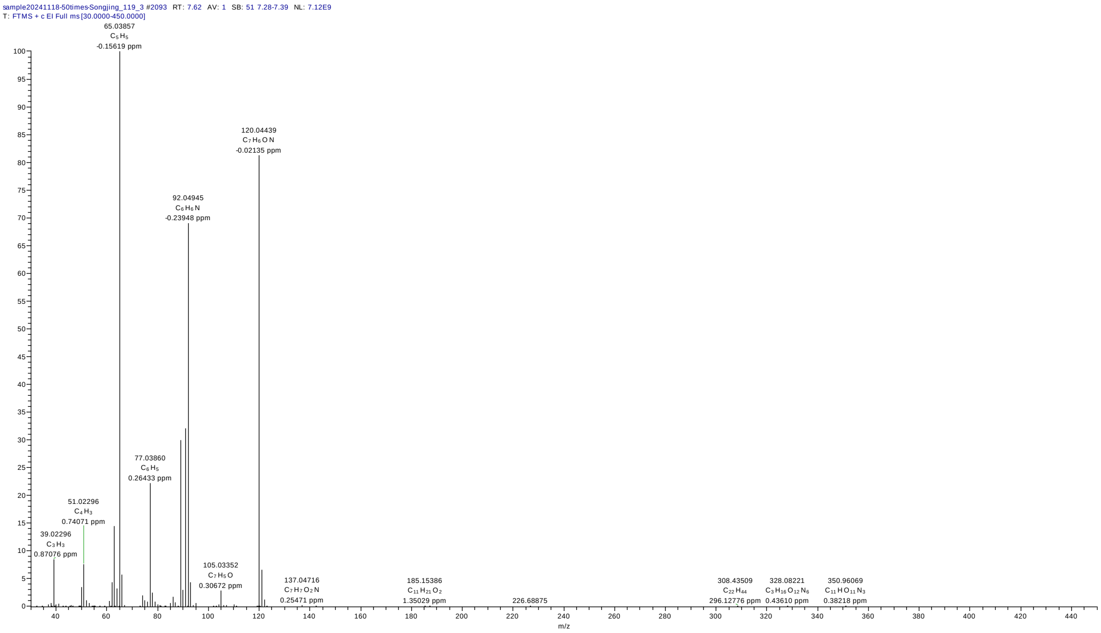

Figure S15. Mass spectrum of *o*-nitrotoluene corresponding to the GC peak at *RT* = 7.66 min.

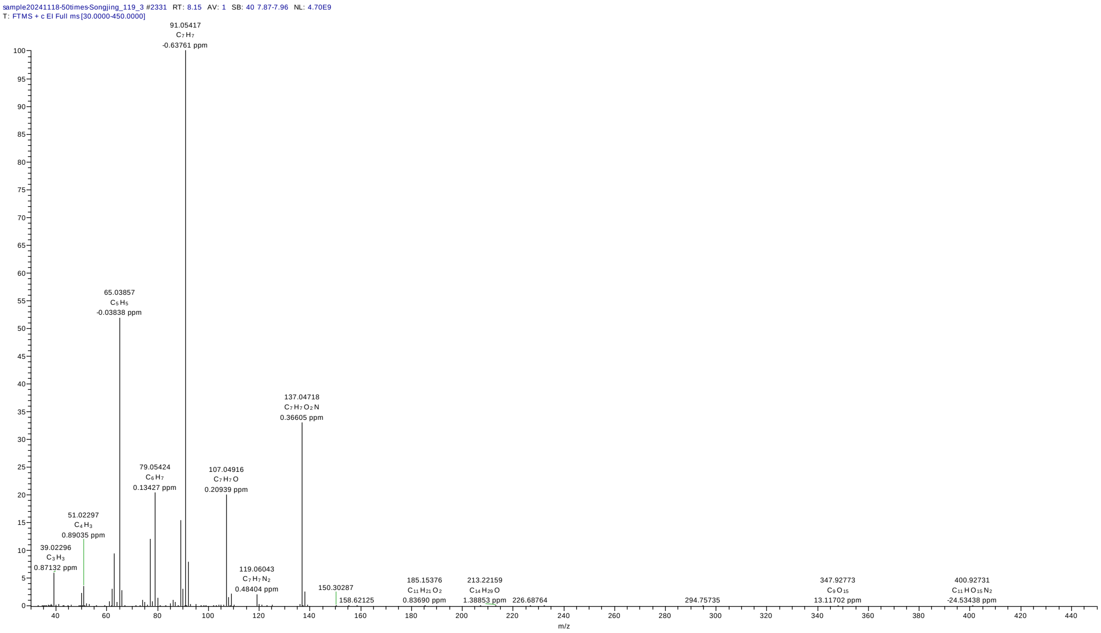

Figure S16. Mass spectrum of *m*-nitrotoluene corresponding to the GC peak at *RT* = 8.15 min.

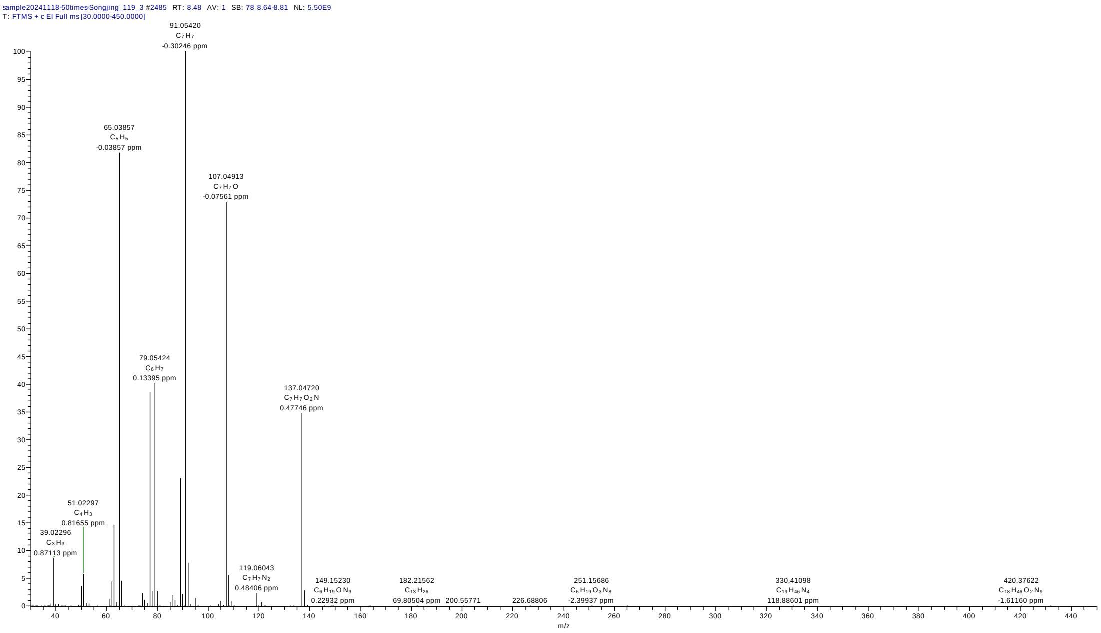

Figure S17. Mass spectrum of *p*-nitrotoluene corresponding to the GC peak at *RT* = 8.52 min.

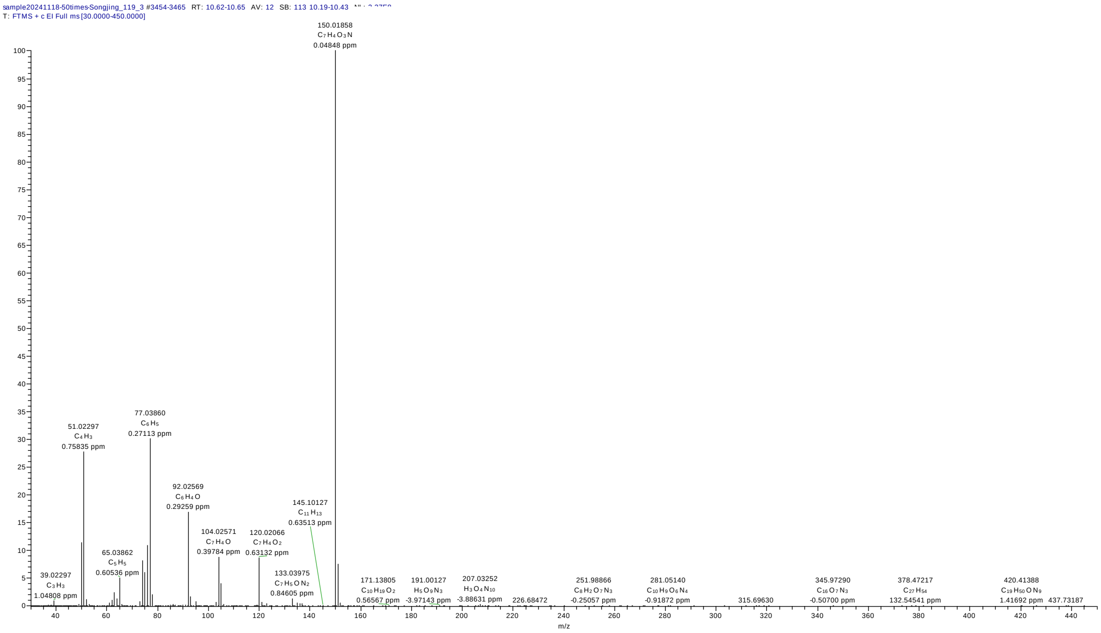

Figure S18. Mass spectrum of *p*-nitrobenzaldehyde corresponding to the GC peak at *RT* = 10.64 min.

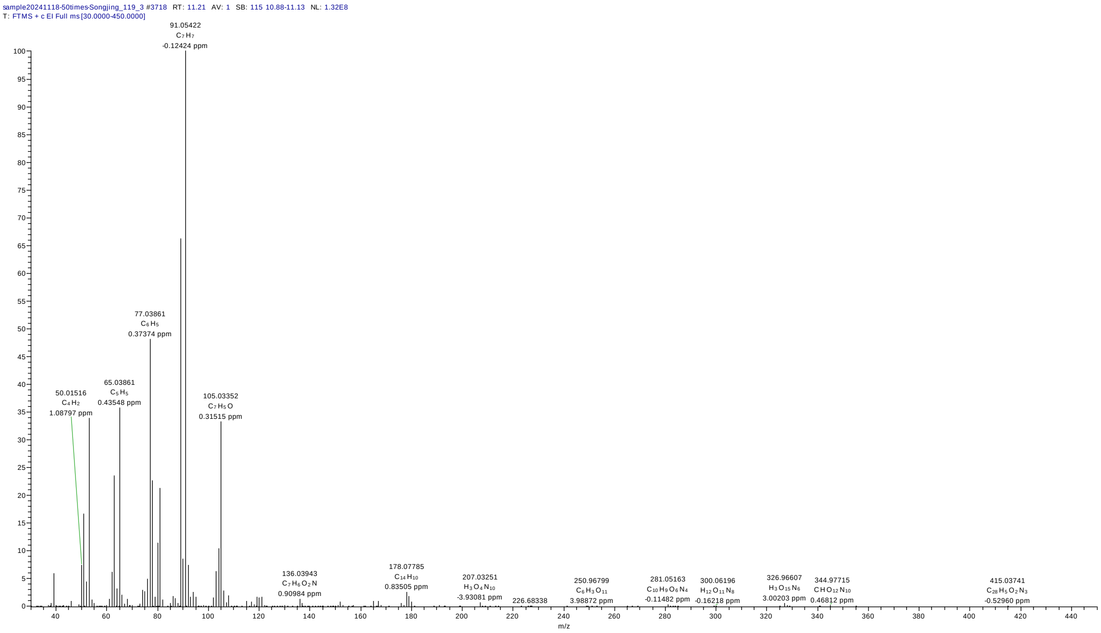

Figure S19. Mass spectrum of *o*-nitrobenzyl alcohol corresponding to the GC peak at *RT* = 11.21 min.

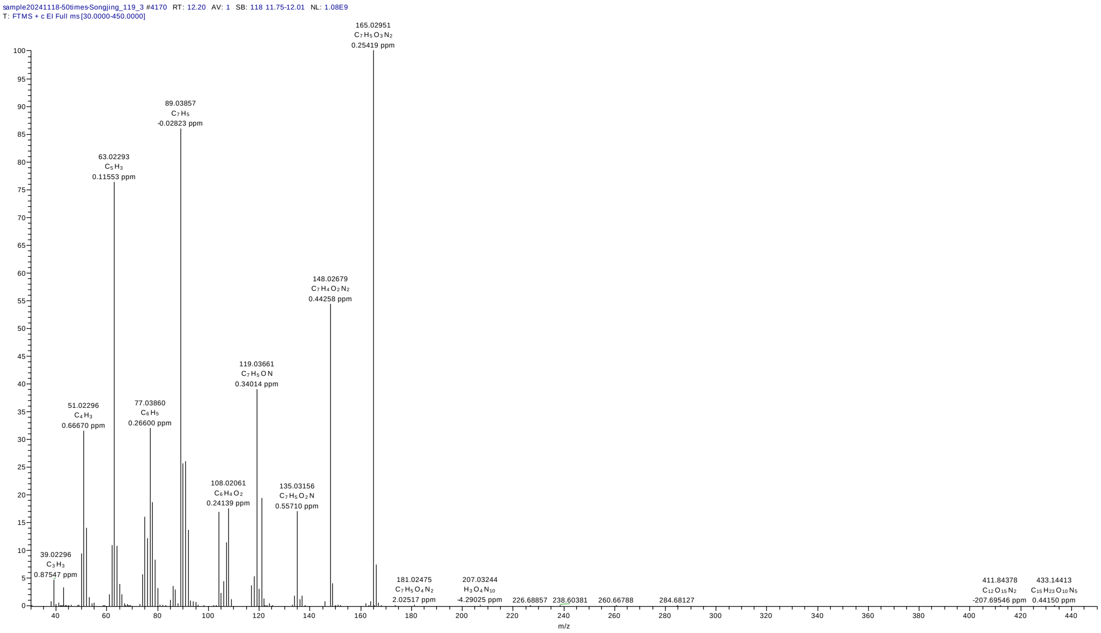

Figure S20. Mass spectrum of 2,6-dinitrotoluene corresponding to the GC peak at *RT* = 12.21 min.

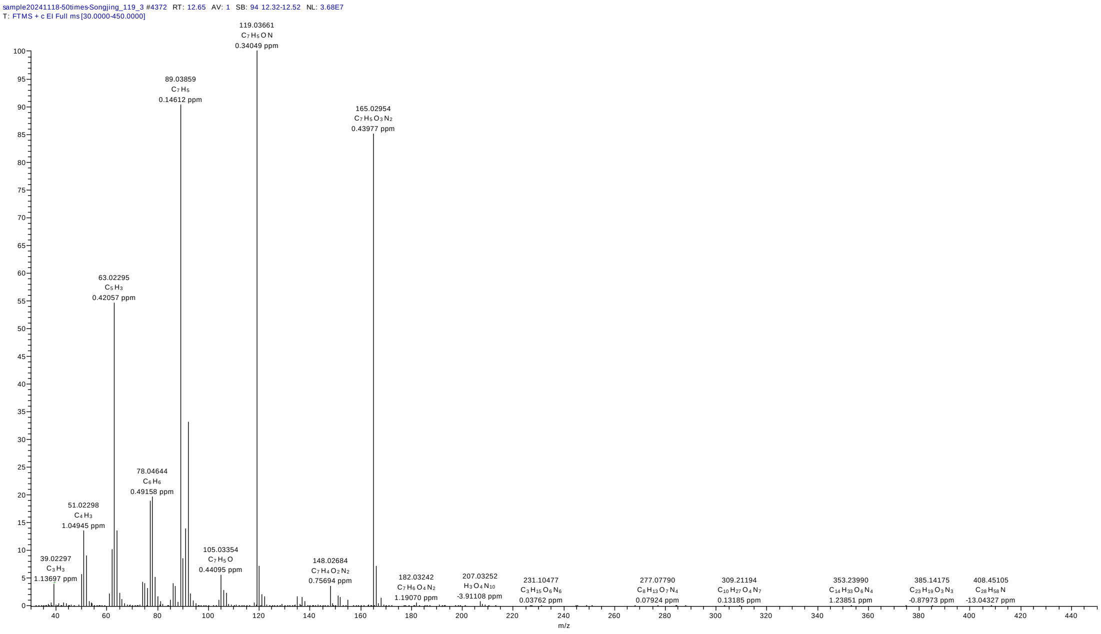

Figure S21. Mass spectrum of 2,5-dinitrotoluene corresponding to the GC peak at *RT* = 12.65 min.

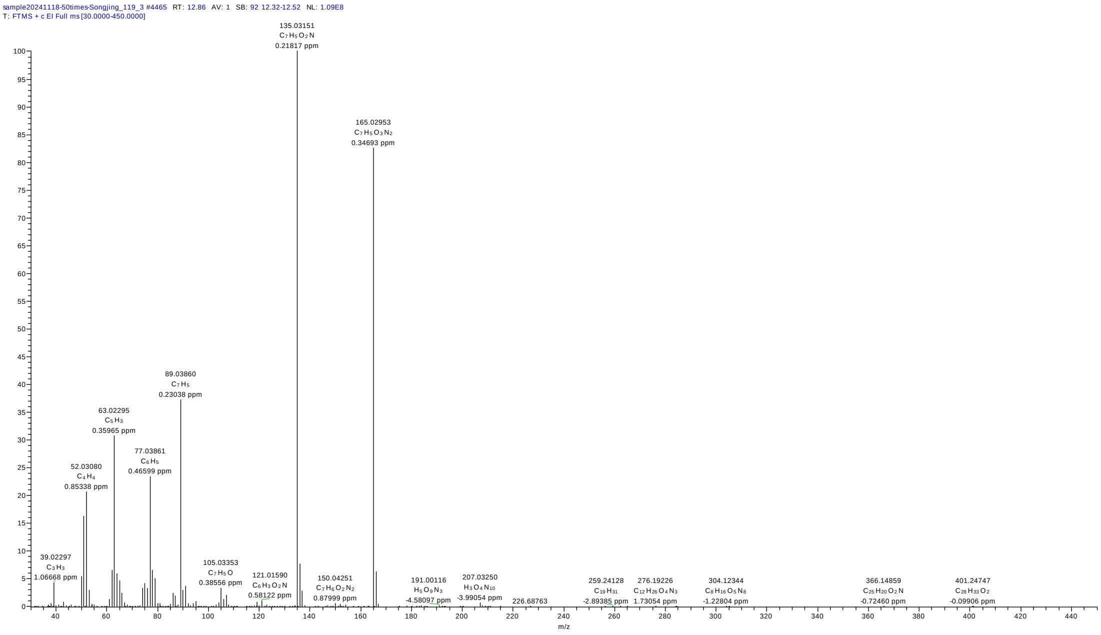

Figure S22. Mass spectrum of 2,3-dinitrotoluene corresponding to the GC peak at *RT* = 12.86 min.

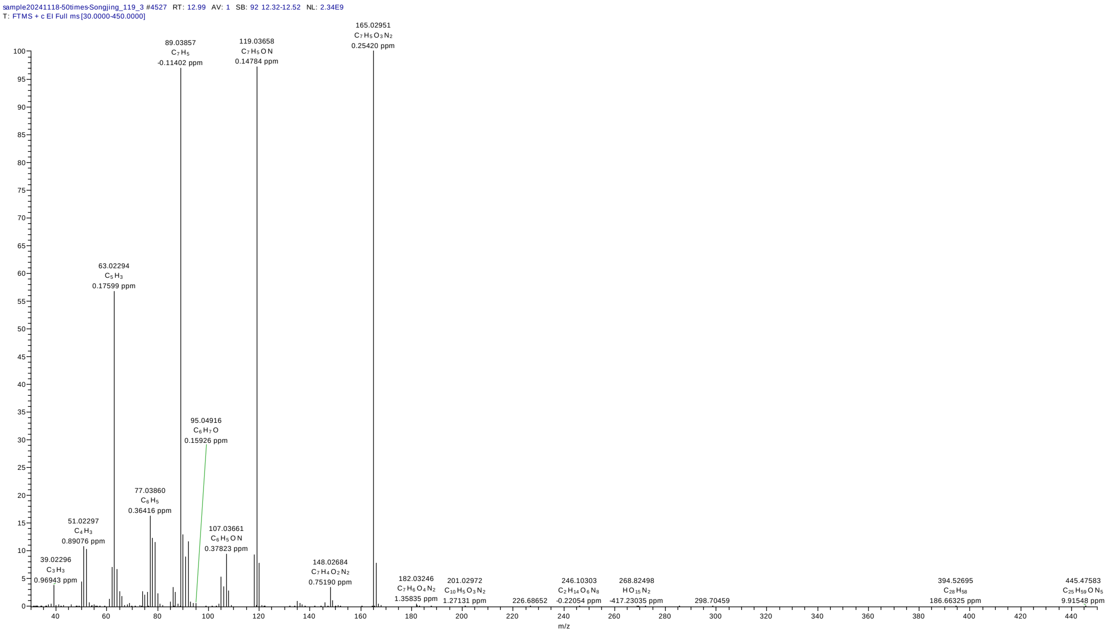

Figure S23. Mass spectrum of 2,4-dinitrotoluene corresponding to the GC peak at *RT* = 12.99 min.

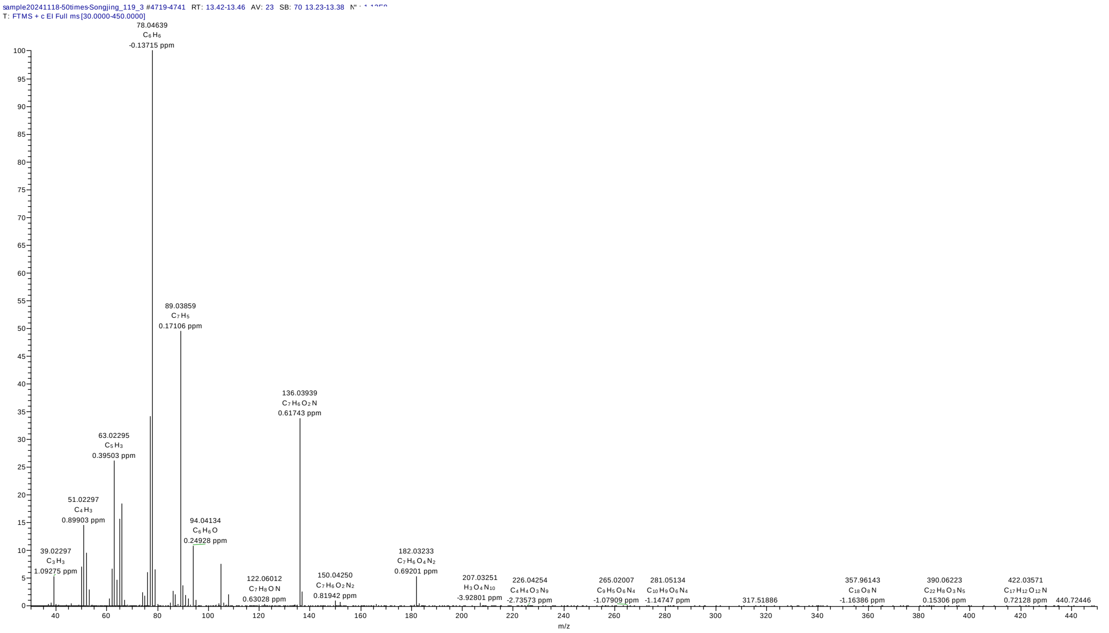

Figure S24. Mass spectrum of 3,4-dinitrotoluene corresponding to the GC peak at *RT* = 13.45 min.

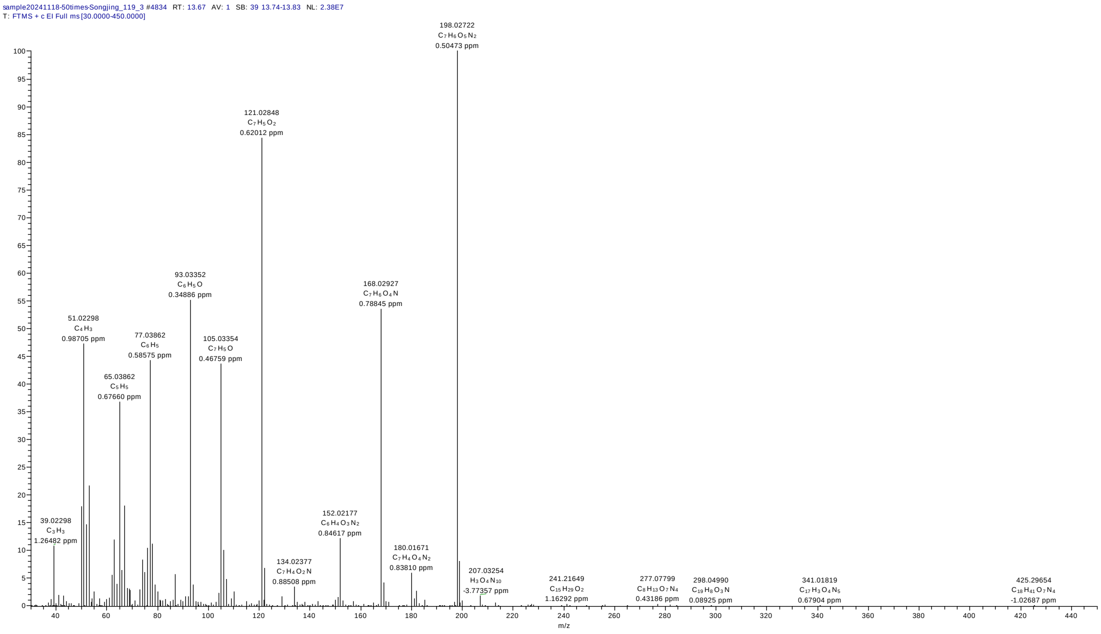

Figure S25. Mass spectrum of 2,4-dinitro-o-cresol corresponding to the GC peak at *RT* = 13.67 min.

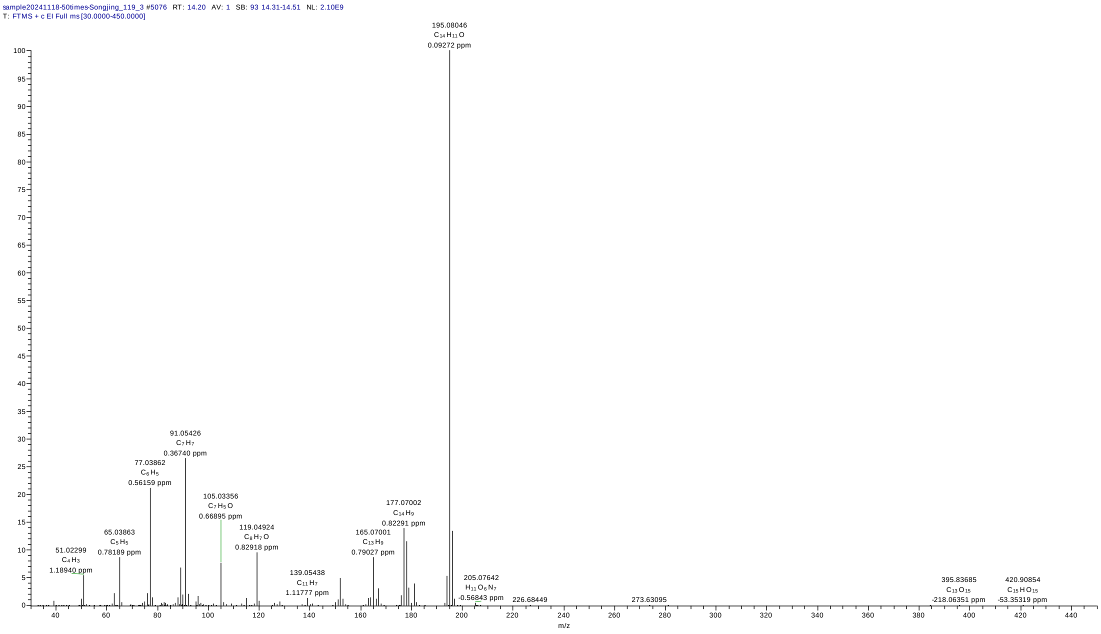

Figure S26. Mass spectrum of 2-methylbenzophenone corresponding to the GC peak at *RT* = 14.21 min.

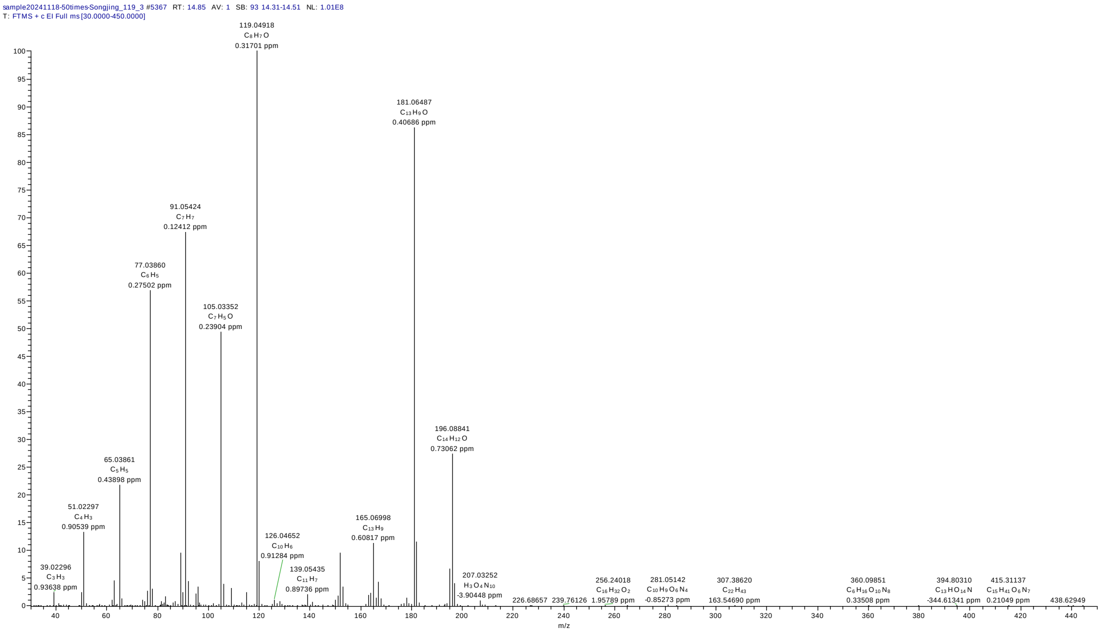

Figure S27. Mass spectrum of 3-methylbenzophenone corresponding to the GC peak at $RT = 14.84$ min.

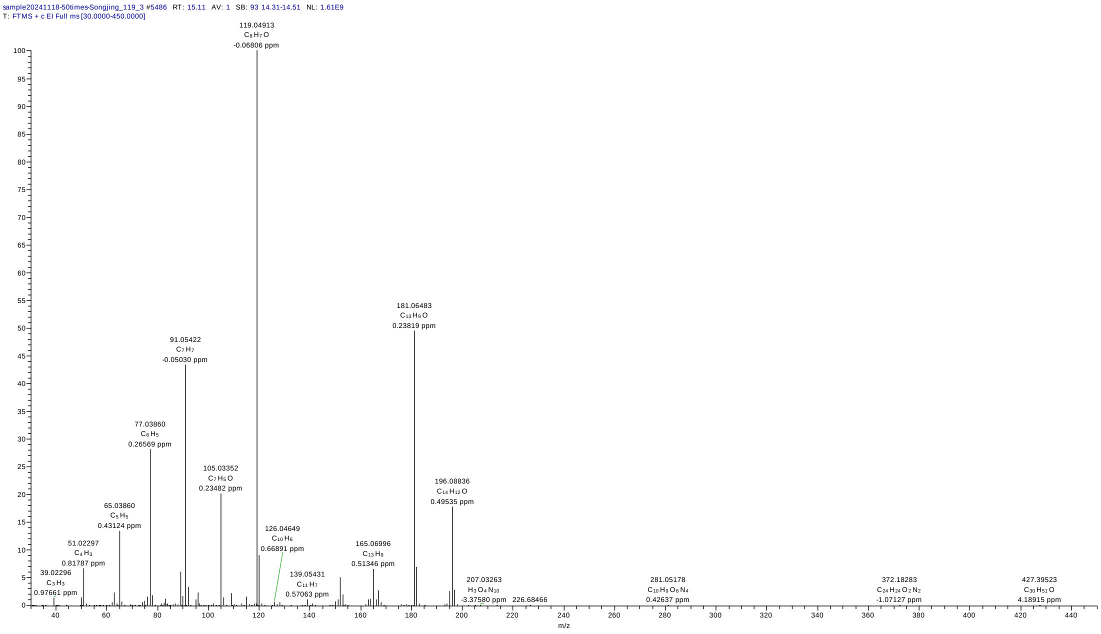

Figure S28. Mass spectrum of 4-methylbenzophenone corresponding to the GC peak at $RT = 15.11$ min.

8.2 Gas chromatography-mass spectrometry (GC-MS) analysis of reaction products in the two-stage countercurrent nitration system

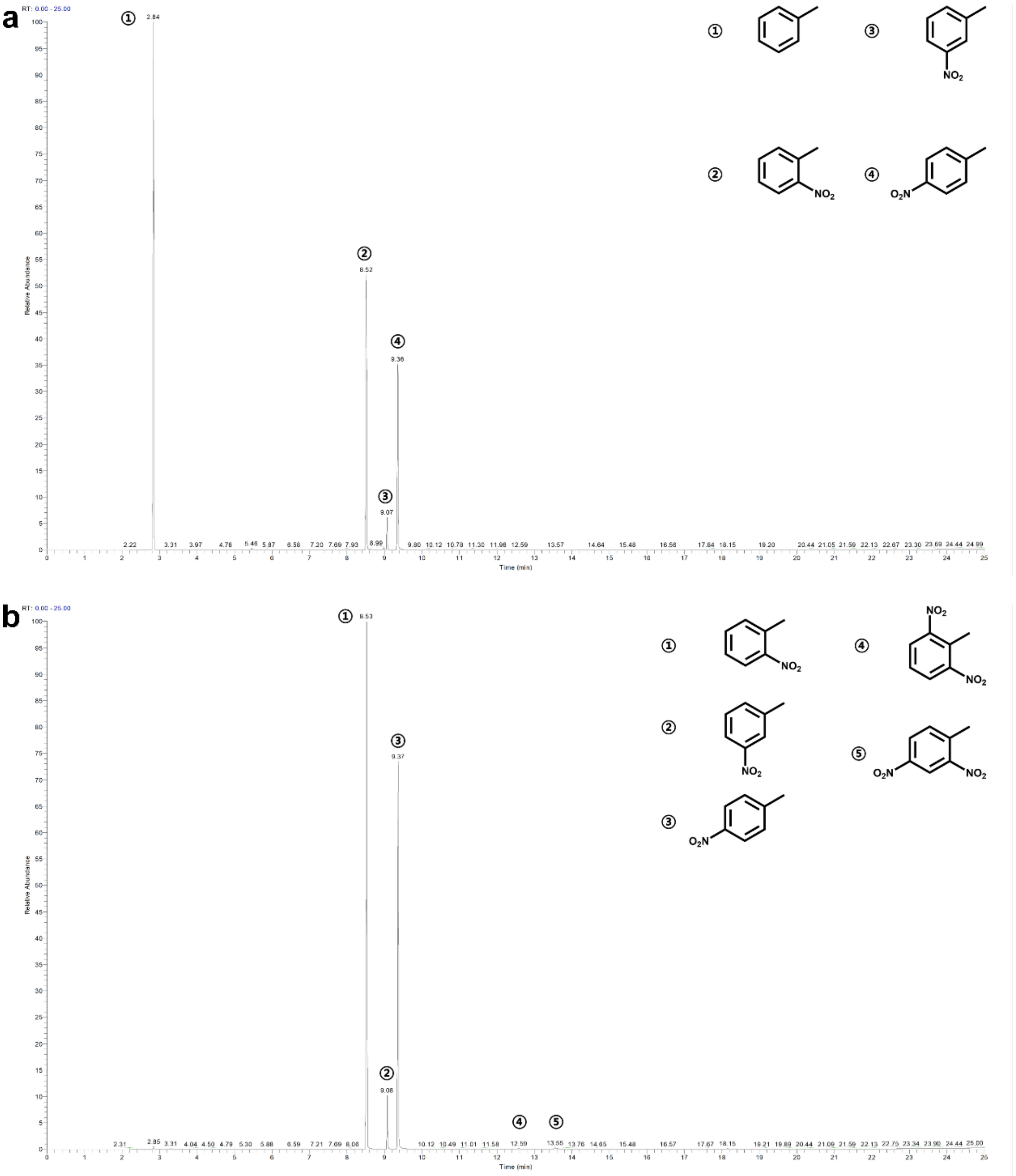

Figure S29. GC-MS spectra of products from the toluene nitration system under two-stage countercurrent mode. a, product from the organic phase of the first-stage microreactor outlet. b, product from the organic phase of the second-stage microreactor outlet.

8.3 Ultra-high performance liquid chromatography-mass spectrometry (UPLC-MS) analysis of reaction products in the two-stage countercurrent nitration system

Toluene was clearly observed in the UPLC chromatogram. However, no corresponding mass spectral signal was detected due to its extremely low ionization efficiency under electrospray ionization (ESI) conditions.

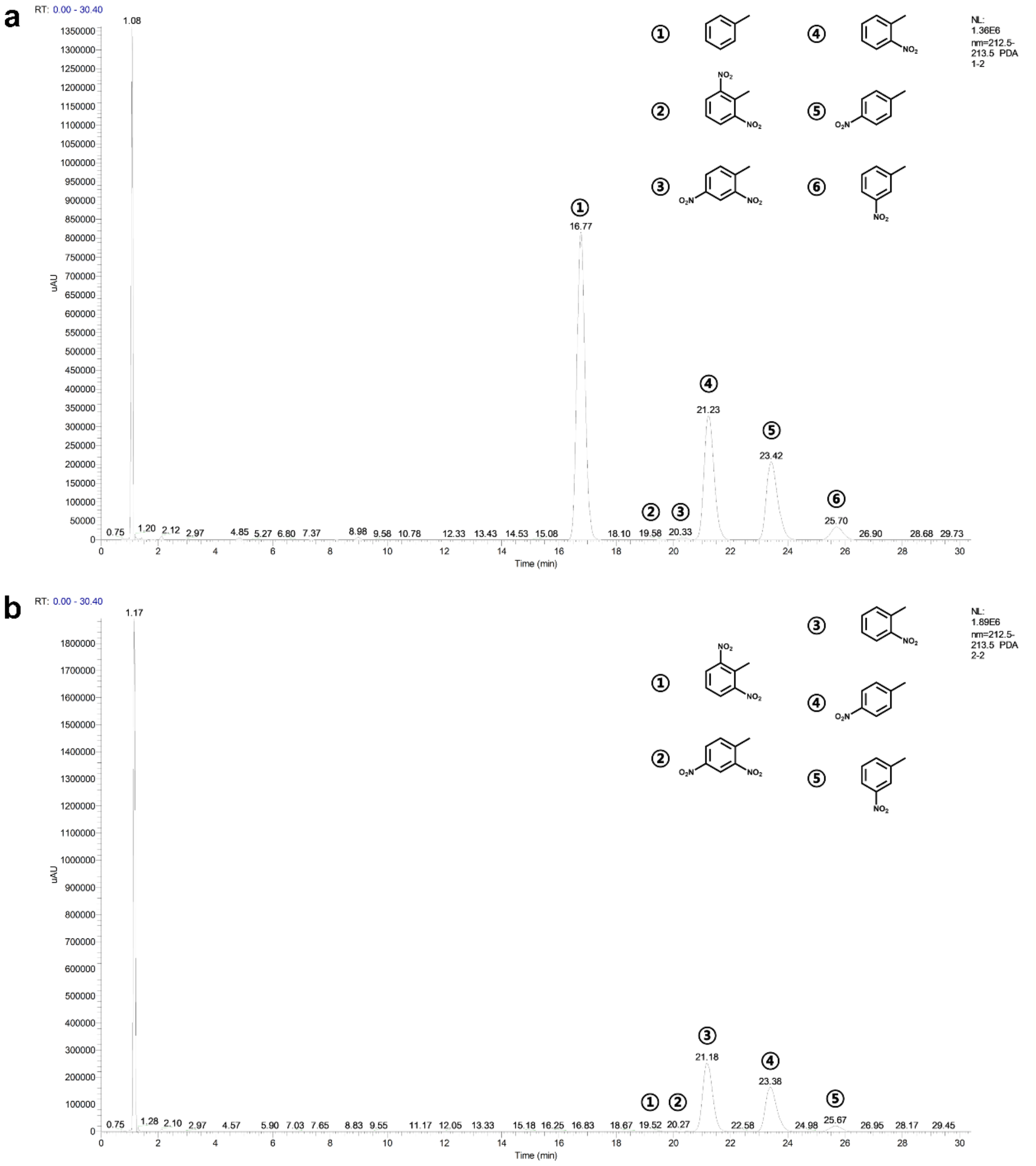

Figure S30. UPLC spectra of products from the toluene nitration system under two-stage countercurrent mode. a, product from the organic phase of the first-stage microreactor outlet. b, product from the organic phase of the second-stage microreactor outlet.

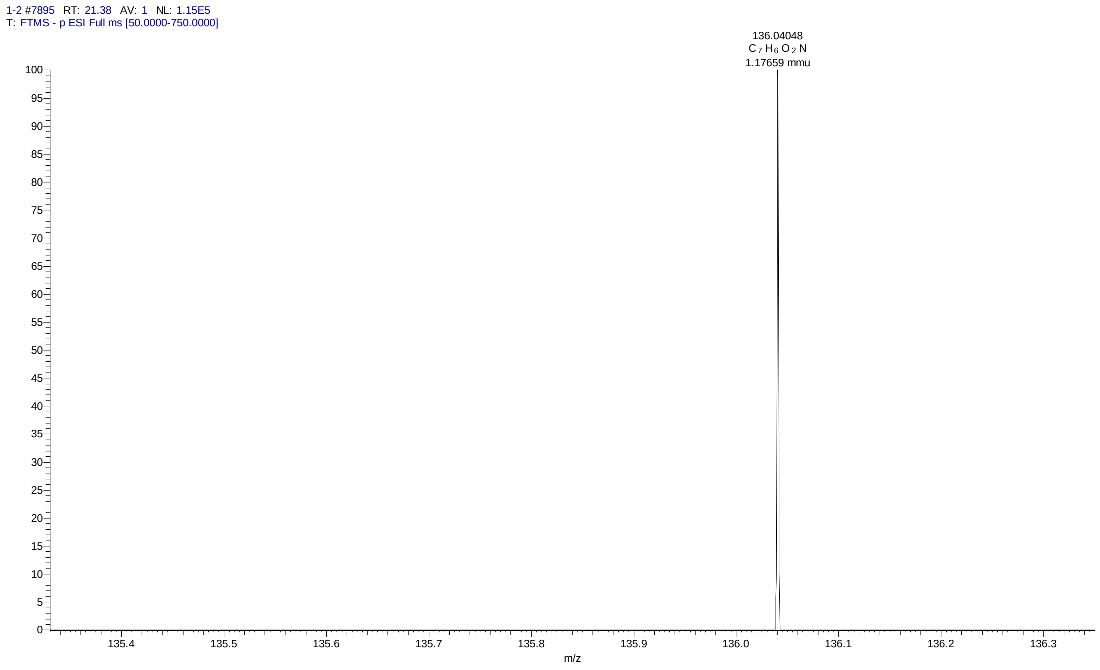

Figure S31. Mass spectrum of *o*-nitrotoluene corresponding to the UPLC peak at *RT* = 21.38 min.

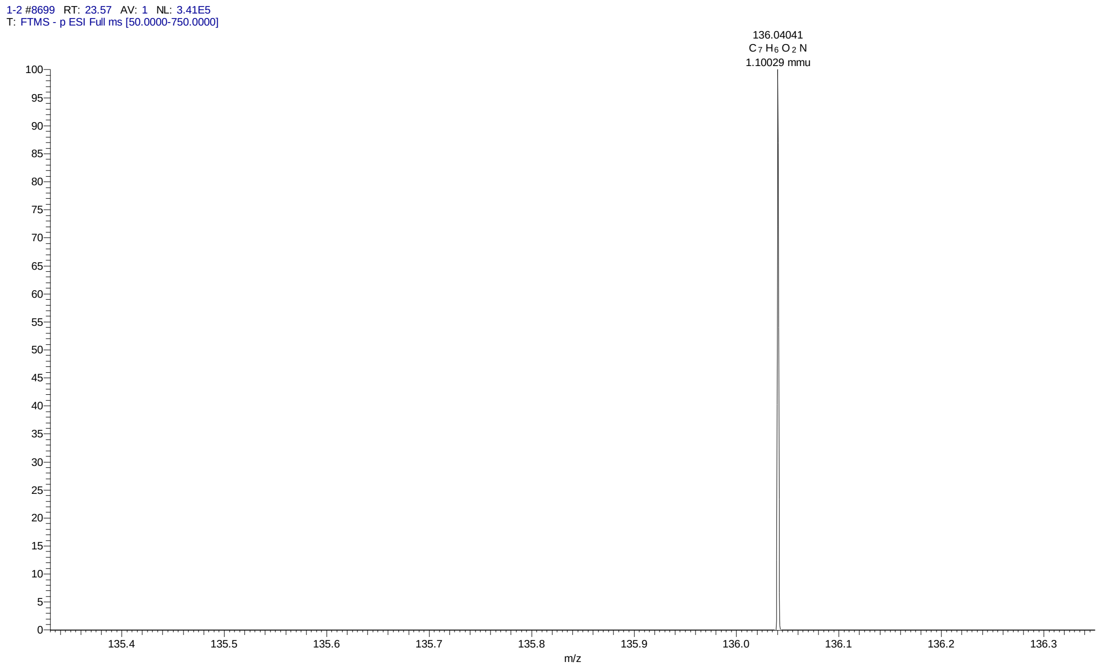

Figure S32. Mass spectrum of *p*-nitrotoluene corresponding to the UPLC peak at *RT* = 23.57 min.

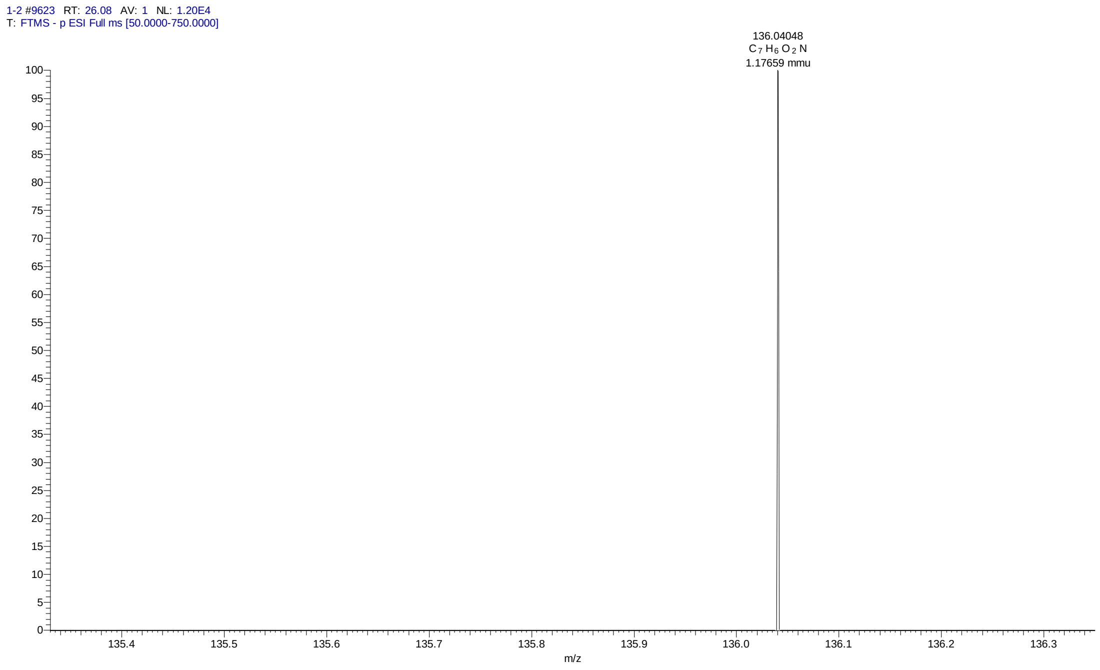

Figure S33. Mass spectrum of *m*-nitrotoluene corresponding to the UPLC peak at RT = 26.08 min.

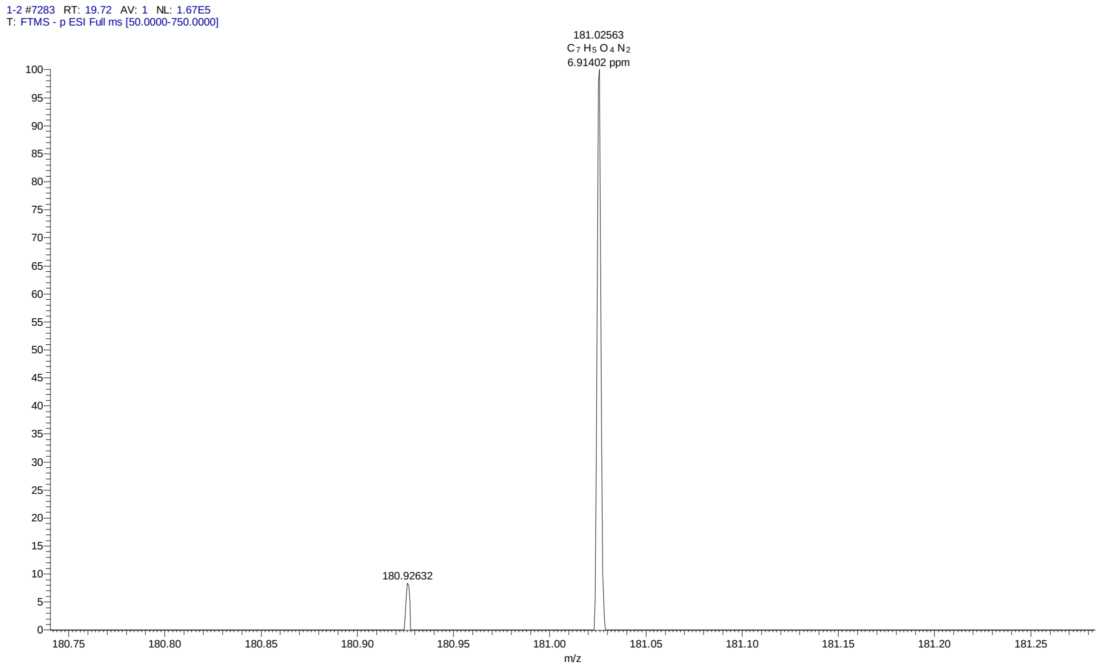

Figure S34. Mass spectrum of 2,6-dinitrotoluene corresponding to the UPLC peak at RT = 19.72 min.

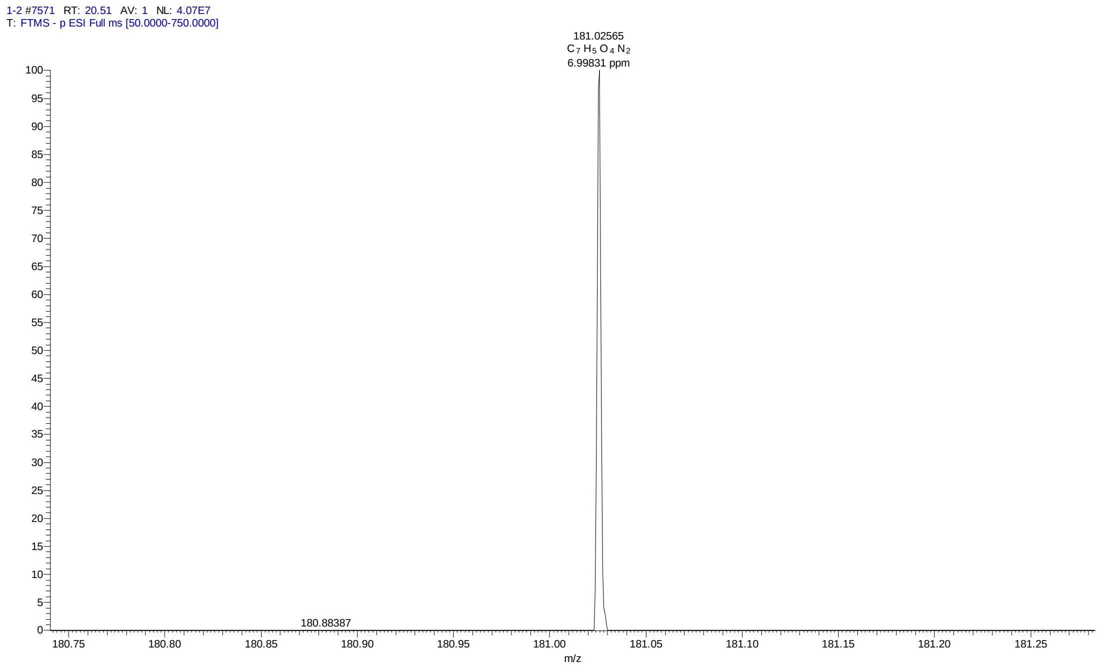

Figure S35. Mass spectrum of 2,4-dinitrotoluene corresponding to the UPLC peak at *RT* = 20.51 min.

Supplementary Note 9: Analysis of Hatta number under actual operating conditions

To evaluate whether the nitration reaction is limited by mass transfer or controlled by intrinsic reaction kinetics under the actual operating conditions, the Hatta number (Ha) was calculated for both stages of the two-stage countercurrent microreaction system.

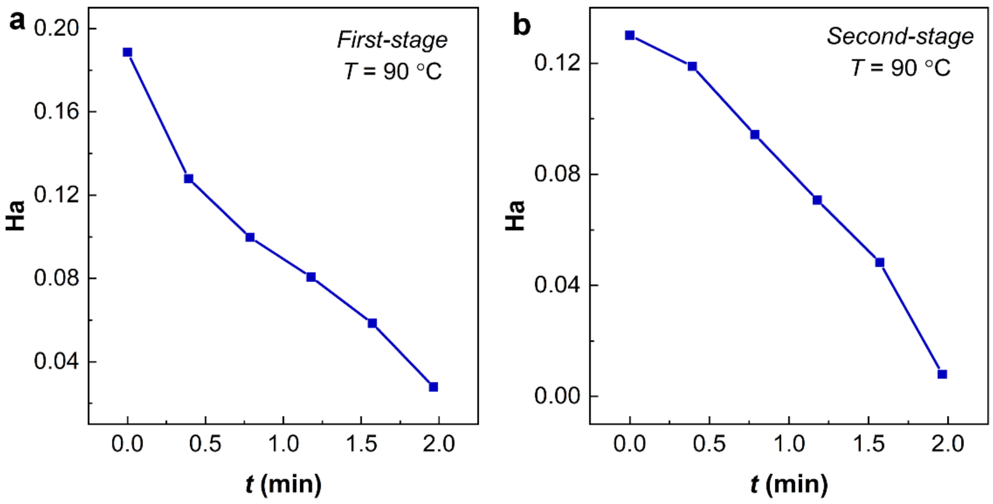

Figure S36. Variation of Hatta number (Ha) with residence time (*t*) in the two countercurrent reaction process. a. Changes in Ha with *t* in the first-stage microreactor. b. Changes in Ha with *t* in the second-stage microreactor.

The Ha number is calculated using the method we previously reported[3]. In general, Ha < 0.3 indicates a kinetically controlled regime, where mass transfer is sufficiently fast and does not limit the overall reaction rate. The calculated Hatta numbers under our actual operating conditions are shown in Figure S36. In both the first-stage and second-stage microreactors, Ha remains consistently below 0.3 throughout the entire residence time range and decreases with increasing residence time. This trend can be attributed to the gradual decrease in reactants and H_2SO_4 (catalyst) concentrations as the reaction proceeds, which reduces the intrinsic reaction rate while the mass transfer capability remains essentially unchanged. These results clearly demonstrate that mass transfer is not rate-limiting under the actual operating conditions employed in this work,

and the reaction is controlled by intrinsic kinetics. Therefore, the enhanced spatiotemporal conversion rate achieved in the two-stage countercurrent mode originates from the optimized reactant concentration distribution along the reactor, rather than from any enhancement of mass transfer. This analysis provides quantitative support for the kinetic optimization strategy proposed in this study.

Supplementary Note 10: Long-term stability test of the two-stage countercurrent microreaction system

A continuous operation test was conducted at 90 °C using toluene nitration as a representative reaction. The system was operated continuously for 10 h under identical reaction conditions to those used in the performance evaluation experiments. During the stability test, samples were collected from the outlet of the second-stage microreactor at regular intervals (every 1 h). The samples were analyzed by gas chromatography to determine toluene conversion (x_T) and DNT selectivity (S_{DNT}). Toluene conversion and DNT selectivity remained stable throughout the entire operation period, fluctuating only within the experimental error range (Figure S37). These indicate that over-nitration and other side reactions did not accumulate with time. No pressure fluctuation, flow instability, or channel blockage was observed during the continuous operation, demonstrating stable hydrodynamic behavior of the two-phase microflow system. These results confirm that the two-stage countercurrent microreaction system maintains excellent stability under strongly oxidizing and corrosive nitration conditions, even at elevated temperature.

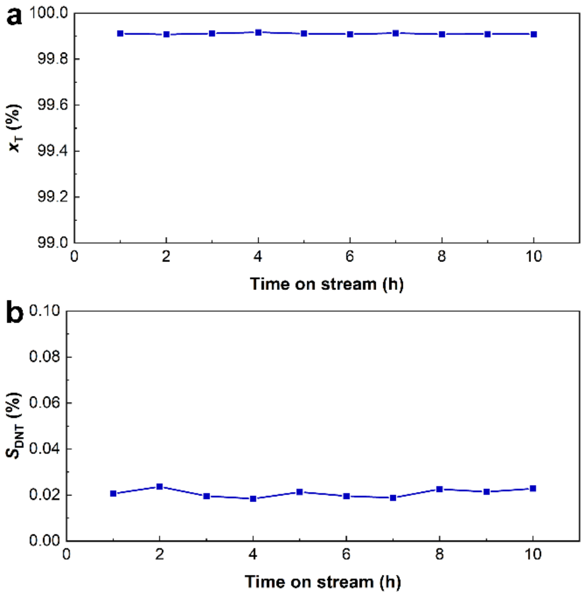

Figure S37. Long-term stability test. a. Toluene conversion as a function of time-on-stream. b. DNT selectivity as a function of time-on-stream.

The potential corrosion of reactor materials under strongly oxidizing and acidic conditions was assessed by inductively coupled plasma optical emission spectroscopy (ICP-OES). Table S7 summarizes the concentrations of representative metallic elements (Fe, Cr, Ni, Cu, and Al) detected in different samples collected along the process.

Sample 1 corresponds to the fresh mixed acid feedstock, while Sample 2 represents the mixed acid after being pumped to the reactor inlet, and Sample 3 corresponds to the aqueous phase collected after reaction. As shown in Table S7, trace amounts of Fe and Cr were detected in Samples 2 and 3, which can be attributed to minor leaching from the 316L stainless-steel pump head used for acid delivery. Importantly, the concentrations of these metal ions remained at the ppm level and showed no significant increase after reaction, indicating that corrosion was minimal and did not accumulate during operation.

Both the microdisperser and the microreactor were fabricated from oleophilic and acid-resistant fluoropolymers. The micro-disperser was made of chlorotrifluoroethylene (CTFE), the microreactor of polytetrafluoroethylene (PTFE), and the phase separator of glass. All of the materials exhibit excellent resistance to the mixed acid of HNO_3 and H_2SO.

Table S7. Concentration of Metal Elements in Different Samples

Sample	Fe (mg/L)	Cr (mg/L)	Ni (mg/L)	Cu (mg/L)	Al (mg/L)
1*	0.04 ± 0.01	0	0	0	0
2*	0.43 ± 0.02	0.05 ± 0.01	0	2.15 ± 0.04	3.29 ± 0.05
3*	0.50 ± 0.04	0.06 ± 0.02	0	1.82 ± 0.11	3.37 ± 0.03

* Sample 1 is a mixed acid raw material. Sample 2 is a mixed acid raw material pumped to the reactor inlet. Sample 3 is the aqueous solution after the reaction.

Supplementary Note 11: Comparison of operating conditions and performance with literature reports

For continuous operation, the spatiotemporal conversion rate (*STCR*) was calculated as:

$$STCR = \frac{\frac{Q_{\mathrm{Ar}}\rho_{\mathrm{Ar}}}{M_{\mathrm{Ar}}} x_{\mathrm{Ar}}}{(Q_{\mathrm{Ar}} + Q_{\mathrm{MA}})t} = \frac{Q_{\mathrm{Ar}}\rho_{\mathrm{Ar}} x_{\mathrm{Ar}}}{(Q_{\mathrm{Ar}} + Q_{\mathrm{MA}}) M_{\mathrm{Ar}} t} \qquad \text{(S19)}$$

Where *STCR* is the spatiotemporal conversion rate of aromatic nitration. Q is the volumetric flow rate. ρ is the density. x is the conversion. M is the molar mass. t is the residence time. Subscript Ar and MA represent the aromatic and mixed acid, respectively.

For batch operation, *STCR* was calculated as:

$$STCR = \frac{n_{\mathrm{ar,0}} x_{\mathrm{ar}}}{Vt} \qquad \text{(S20)}$$

Where $n_{\mathrm{ar,0}}$ is the initial moles of aromatic. V is the batch reactor volume. t is the reaction time.

Table S8 summarizes the operating conditions and reaction performance of toluene nitration reported in representative literature studies, together with the single-stage co-current and two-stage countercurrent processes developed in this work.

Table S8. Comparison of operating conditions and reaction performance for toluene nitration reported in the literature and in this work.

Authors (Year)	Reaction mode	V (mL)	$w_{H2SO4,0}$ (%)	M	T (°C)	t (min)	x_T (%)	S_{MNT} (%)	$STCR$ (mol/L·min)
Panke et al. (2003)[4]	Batch	25	62	1.5	65	140	100	94.6	1.3×10^{-5}
Burns et al. (2002)[5]	Continuous	0.0238	80	HNO_3 excessive	RT*	0.2	80%	63.3	6.3×10^{-3}
Panke et al. (2003)[4]		1.2	62	1.5	65	15.0	98.8	89.5	3.1×10^{-4}
Fu et al. (2022)[6]		132	—	1.2	45	2.2	86	95	1.7×10^{-4}
Yang et al. (2022)[7]		10	58	1.3	49	2.8	85	91.3	1.7×10^{-3}
Han et al. (2024)[8]		6.74	80	1.4	70	1.2	100	91.5	6.3×10^{-3}
Han et al. (2024)[8]		6.65	79	1.3	80	1.0	100	89.9	7.5×10^{-3}
Han et al. (2024)[8]		6.82	78	1.4	70	1.2	99.1	90.6	6.2×10^{-3}
Single-stage co-current (This work)		4	71.5	1.01	90	3.9	69	99.1	0.8×10^{-3}
Two-stage countercurrent (This work)		4	71.5	1.01	90	3.9	99.9	99.8	1.2×10^{-3}

RT* means room temperature.

Supplementary Note 12: Determination of the optimal H_2SO_4/HNO_3 dilution window

To evaluate the effect of acid composition on reaction performance and to define an optimal dilution window, the effect of the H_2SO_4/HNO_3 molar ratio (n_{H2SO4}/n_{HNO3}) on toluene nitration was investigated over a wide range, spanning from fuming HNO_3 (n_{H2SO4}/n_{HNO3}=0) to H_2SO_4-dominated mixed acids which commonly employ in conventional aromatic nitration processes (n_{H2SO4}/n_{HNO3}>5) (Figure S38).

When fuming HNO_3 was used as the nitrating agent (n_{H2SO4}/n_{HNO3}=0), the reaction exhibited a relatively low overall conversion, accompanied by an increase in over-nitration selectivity. Although both toluene and MNT exhibit negligible solubility in fuming HNO_3, the extremely high initial HNO_3 concentration leads to a very rapid initial reaction and intense local heat release, resulting in a substantial interfacial temperature rise. Given that the activation energy of over-nitration is higher than that of mononitration, it favors the over-nitration reaction. As the reaction proceeds, the in-situ generated H_2O rapidly dilutes HNO_3, suppressing the formation of the active nitrating species (NO_2^+), which limits the achievable conversion.

Upon introducing H_2SO_4, H_2SO_4 acts as a catalyst and a dehydrating agent, efficiently absorbing the water produced during mononitration and thereby sustaining NO_2^+ generation. As a result, the reaction rate increases compared with the fuming HNO_3 system. However, when the H_2SO_4 dosage is too low (0 <n_{H2SO4}/n_{HNO3}< 0.7), the nitration system remains dominated by HNO_3. In this regime, the initially high HNO_3 concentration leads to an intense reaction at the early stage, which kinetically promotes over-nitration side reactions. Meanwhile, the generated H_2O rapidly dilutes the H_2SO_4, weakening its catalytic and dehydrating roles and suppressing sustained NO_2^+ formation, ultimately resulting in both lower conversion and selectivity of MNT.

Conversely, at high H_2SO_4 dosages (n_{H2SO4}/n_{HNO3}>1.5), the product inhibition mechanism becomes ineffective. Under these conditions, the high H_2SO_4 concentration significantly enhances the solubility of nitroaromatic products in the acid phase,

facilitating their dissolution and over-nitration side reaction. By considering conversion and suppression of over-nitration, an optimal dilution window is identified at $0.7<n_{H2SO4}/n_{HNO3}<1.5$. Within this range, both high conversion and high mononitration selectivity can be achieved simultaneously. This optimal dilution window reflects a balance between maintaining sufficient nitrating strength and enabling the product inhibition mechanism to suppress over-nitration.

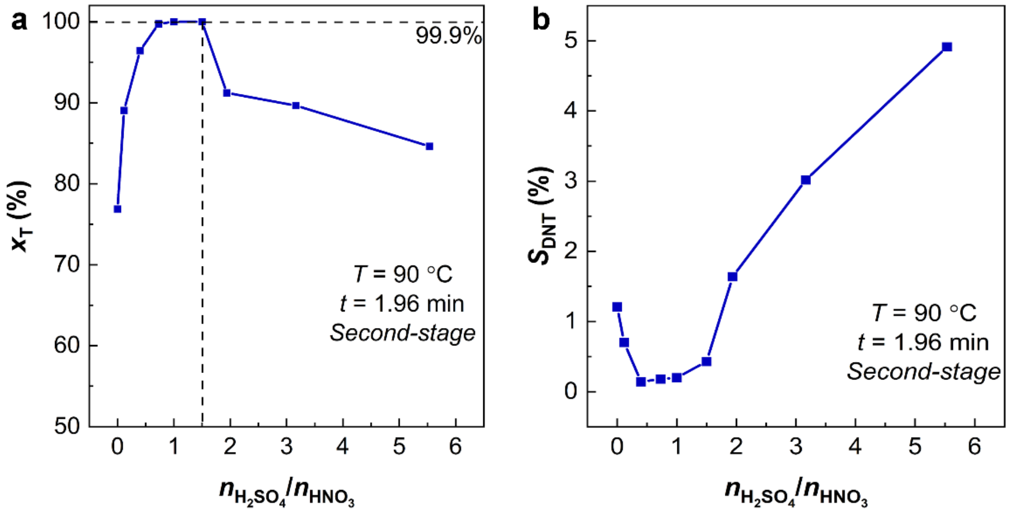

Figure S38. Effect of molar ratio of H_2SO_4 to HNO_3 (n_{H2SO4}/n_{HNO3}) on reaction performance. a. Effect of n_{H2SO4}/n_{HNO3} on toluene nitration. b. Effect of n_{H2SO4}/n_{HNO3} on DNT selectivity.

References

1. Zhao W*, et al.* Safe, Green, and Efficient Synthesis of m-Dinitrobenzene via Two-Step Nitration in a Continuous-Flow Microreactor. **8**, e202204997 (2023).
2. Han B-C, Chen Y-D, Zou H-W, Yu G-G, Sheng C, Wang G-Z. Study on characteristics of toluene/chlorobenzene nitrification in different microreactors. *Chemical Engineering Research and Design* **205**, 343-353 (2024).
3. Song J, Cui Y, Wang Y, Wang K, Deng J, Luo G. Accurate determination of the kinetics of toluene nitration in a liquid–liquid microflow system. *Journal of Flow Chemistry* **13**, 311-323 (2023).
4. Panke G, Schwalbe T, Stirner W, Taghavi-Moghadam S, Wille G. A Practical Approach of Continuous Processing to High Energetic Nitration Reactions in Microreactors. *Synthesis* **2003**, 2827-2830 (2003).
5. Burns JR, Ramshaw C. A Microreactor for the Nitration of Benzene and Toluene. *Chemical Engineering Communications* **189**, 1611-1628 (2002).
6. Fu G, Ni L, Wei D, Jiang J, Chen Z, Pan Y. Scale-up and safety of toluene nitration in a meso-scale flow reactor. *Process Safety and Environmental Protection* **160**, 385-396 (2022).
7. Yang A*, et al.* Experimental investigation of mononitrotoluene preparation in a continuous-flow microreactor. *Research on Chemical Intermediates* **48**, 4373-4390 (2022).
8. Han B, Chen Y, Zou H, Yu G, Sheng C, Wang G. Study on characteristics of toluene/chlorobenzene nitrification in different microreactors. *Chemical Engineering Research and Design* **205**, 343-353 (2024).
